# Ethical analysis of the European normative framework on fertility preservation

**DOI:** 10.1186/s12910-026-01393-8

**Published:** 2026-02-02

**Authors:** Silviya Aleksandrova-Yankulovska, Marcin Orzechowski, Katharina Hancke, Karin Bundschu, Florian Steger

**Affiliations:** 1 Institute of the History, Philosophy and Ethics of Medicine, Ulm University, Oberberghof 7, Ulm, 89081 Germany; 2 Department of Gynaecology and Obstetrics, Ulm University, Prittwitzstr. 43, Ulm, 89075 Germany

**Keywords:** Fertility preservation, European regulation, Guidelines, Legal documents, Principles of bioethics

## Abstract

**Background:**

Fertility preservation involves storing reproductive tissues or cells to enable reproduction later in life. It is often part of assisted reproduction but is also used independently. Our research aim is to study the European normative framework of fertility preservation in view of identifying and ethically analysing the main topics of regulation.

**Methods:**

A systematic literature search in EUR-Lex, national legal databases, Science Direct, Web of Science, PubMed, and a subsequent thematic and ethical analysis were performed. Altogether, 63 documents, including 37 law-informing documents and 26 guidelines, were analysed.

**Results:**

Ten thematic topics were identified: (I) definitions of fertility preservation; (II) age limits for fertility preservation; (III) type of preserved reproductive biomaterial; (IV) posthumous usage; (V) informed consent; (VI) state/health insurance funding; (VII) storage period; (VIII) social egg freezing; (IX) requirements for partnership status; (X) special provisions for transgender individuals. These topics were assessed against the four bioethical principles. Legal norms mostly reflected the principle of justice, while guidelines focused on beneficence. Among all topics, the most permissively regulated were social egg freezing, partnership status, and fertility preservation for transgender individuals. In contrast, age limits were the most strictly regulated. Overall, the European framework seeks to balance equal access for diverse groups - such as single women, couples, and transgender individuals - with the aim to keep reproduction within traditionally accepted age limits.

**Conclusion:**

Laws and guidelines dealing with fertility preservation were not equally inclusive for all user groups and they did not provide sufficient guidance on ethically challenging aspects. Our focus was on identifying and ethically analysing common gaps and inconsistencies in the European normative framework rather than suggesting concrete legal changes. The ethical analysis, based on the four principles of bioethics, enabled us to make concrete suggestions for improving the guidelines - particularly by adding ethical content related to the studied topics.

**Supplementary Information:**

The online version contains supplementary material available at 10.1186/s12910-026-01393-8.

## Introduction

Fertility holds a unique place in human life. It is deeply connected to private experiences and existential meaning. Infertility is not solely a medical condition, but it significantly affects the quality of life [1]. Despite the big advancement of artificial reproductive technologies (ART), the ethical guidance in fertility care stays behind. Clinical success often comes first. Yet reproductive decisions involve complex emotional, social, and moral dimensions [2]. Given the centrality of reproduction to human life, ethical considerations should be integrated more thoroughly into clinical decision-making.

The preservation of reproductive tissues and cells is a key part of assisted reproductive technologies. It can also be used separately to help preserve fertility. Several methods are available, such as cryopreservation of gametes and reproductive tissues, ovarian transposition, and ovarian suppression [3]. In this study, we use the term “fertility preservation” as defined in EU regulation: “the process of saving or protecting a person’s reproductive substance of human origin intended to be used later in that person’s life” for medically assisted reproduction [4]. Reproductive substance of human origin means human sperm, oocytes, ovarian and testicular tissue [4]. Embryos are also considered reproductive substance of human origin even though they are not collected from the human body [4]. However, we did not extend our study to embryo freezing regulations.

Fertility preservation began as a solution for cancer patients and those receiving gonadotoxic treatments. Since then, it has become a fast-growing area in reproductive medicine [5], with broader applications. Today, new groups seek access to fertility preservation. These include healthy women who want to extend their reproductive span, transgender individuals, and women with endometriosis.

While assisted reproductive technologies are highly regulated, fertility preservation as a separate service often lacks clear legal oversight. Regulation is missing or the legal norms are underdeveloped for certain patient groups, such as single women and transgender individuals [6]. There is still limited research on how well current rules address ethical issues in fertility preservation. It remains unclear whether existing laws and guidelines offer enough support for dealing with difficult ethical questions in practice.

This research gap has led us to explore the European normative framework for fertility preservation. We study laws, guidelines, and regulations that shape how these services are provided.

Although these terms are widely used, a brief clarification is needed. Laws (hard norms) are created, amended, or repealed by legislative bodies. Executive authorities are responsible for enforcing these laws, often by developing regulations. Regulations provide more detailed rules and are adopted through a formal process. Once approved, they carry the force of law. Guidance, by contrast, includes materials like guidelines, recommendations, position statements, and consensus papers (soft norms). These are published by professional bodies to support the interpretation of legal rules and guide practice. While helpful in decision-making, they are not legally binding [7, 8] contrary to the laws that are binding.

Our research focuses on the European normative framework of fertility preservation - laws, regulations, and guidelines. We aim to identify the main topics of regulation and to ethically analyse them through the prism of the four bioethics principles [9]. To this end, we examine various sources to clarify normative boundaries and related ethical challenges, seeking to determine whether existing laws and guidelines provide sufficient support for addressing complex ethical questions in fertility preservation. Finally, we aim to provide argumented recommendations for improvement of guidelines on fertility preservation.

## Methods

A systematic search was conducted using primary and secondary literature sources. The search followed predefined inclusion and exclusion criteria.

### Search strategy

Primary sources included EU legislation from the EUR-Lex platform and national legal databases. The search algorithm used the keywords: “oocytes” OR “sperm” OR “ovarian tissue” OR “testicular tissue” AND “freezing” OR “fertility preservation”.

Secondary sources were scientific articles that discussed national laws not directly accessible. These were searched in PubMed, Web of Science, and Science Direct using the keywords “fertility preservation” AND “law”.

Guidelines were searched using the same databases with the keyword combination “fertility preservation” AND “guideline”.

#### Inclusion and exclusion criteria

The inclusion criteria were: specific reference to fertility preservation; English language; European context; published between 2014 and 2024; full text available; legal issues clearly addressed; and laws currently in force.

The exclusion criteria were: no specific mention of fertility preservation; no focus on regulation issues; no link to humans; only clinical content; and laws not in force.

### Data analysis

All identified documents were analysed thematically by the first author to uncover the main themes related to fertility preservation. The results were discussed between all members of the authorship team. Thematic analysis is a qualitative research approach used to identify and analyse common and recurring themes in text sources [10]. Next, we conducted an ethical analysis based on the four principles of bioethics: autonomy, beneficence, non-maleficence, and justice [9]. The four principles approach was chosen because of its wide application in medical ethics and usefulness for mixed audience of healthcare professionals, legal and bioethics experts.

## Results

In the primary literature search, 45 documents were initially identified. Four were duplicates. Twenty-nine did not meet the inclusion criteria. Two documents could not be retrieved due to the lack of an English version. In the end, 10 primary legal sources were considered eligible.

The search for secondary sources returned 452 records. After removing 32 duplicates, titles and abstracts were screened. A total of 390 records were excluded for not meeting the criteria. Thirty publications were marked as relevant. After full-text screening, 3 more were excluded. This left 27 secondary legal sources for analysis.

**Fig. 1:**
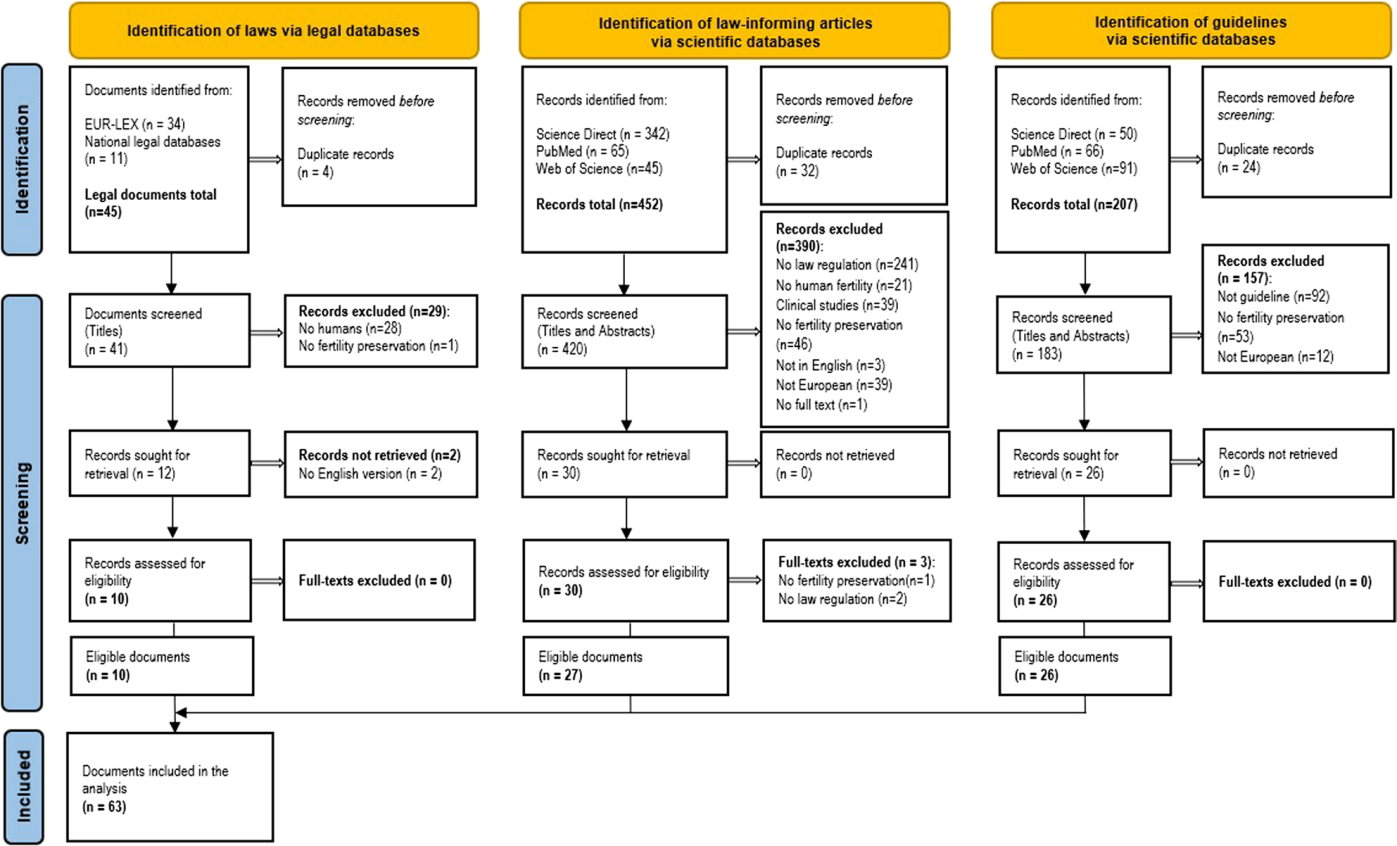
Flow chart of document search

The search for guidelines identified 207 records. After removing 24 duplicates and excluding 157 that did not meet the inclusion criteria, 26 documents were retrieved in full (Fig. 1). These included guidelines, recommendations, position statements, and consensus criteria. For consistency, we refer to all of them as guidelines in this paper.

In total, 63 documents were analysed: 37 law-informing documents and 26 guidelines (Additional file 1 in Supplementary Material).

The documents covered 44 European countries and included 47 laws. To present a full picture of the European normative framework on fertility preservation, we referred to the United Nations list of European countries [11] and the Council of Europe member list [12]. No data were found for Andorra, Azerbaijan, Liechtenstein, Monaco, San Marino, and the Holy See.

The regulation of fertility preservation was part of laws in the following areas: laws about ART − 29; laws about health services − 5; health insurance laws − 1; laws on bioethics − 1; general health acts − 10; laws on genetic integrity − 1.

The 26 analysed guidelines were issued by different professional organisations as follows: international bodies − 16 guidelines; French bodies − 3; German bodies − 2; British bodies − 2; Spanish bodies − 2, and 1 body from Bosnia and Herzegovina. In regard to the subject area, the guidelines were distributed as follows: cancer patients − 15 guidelines; fertility preservation − 7; patients with endometriosis − 1; transgender people − 1; patients with systemic lupus erythematosus − 1; general fertility problems − 1.

### Thematic analysis

The thematic analysis of laws identified 10 topics related specifically to fertility preservation: Topic I – definitions of fertility preservation; Topic II - age limits for fertility preservation/ART access; Topic III – type of preserved reproductive biomaterial; Topic IV - posthumous usage; Topic V - informed consent; Topic VI - state/health insurance funding; Topic VII - storage period; Topic VIII - social egg freezing; Topic IX - requirements for partnership status; Topic X - special provisions for transgender individuals.

The guidelines were studied through the prism of the same 10 identified topics. This allowed comparison with laws and created a full picture of the normative framework for fertility preservation.

Detailed results of the thematic analysis of laws are in Additional file 2 in Supplementary Material. Detailed results for guidelines are in Additional file 3 in Supplementary Material.

Regarding Topic I – definitions of fertility preservation, particular definitions were found in 1 law and 1 guideline:

1. “Fertility preservation - cryopreservation of reproductive tissues or cells to preserve their reproductive capacity. The process of saving or protecting a person’s oocytes, sperm, and/or reproductive tissue (ovarian tissue, testicular tissue) so that they can use them to try to have biological children later in life.” [13].
2. “Fertility preservation techniques will be performed in patients with a possible risk of loss of their reproductive capacity associated with exposure to gametotoxic treatments or pathological processes with a proven risk of premature ovarian failure or primary testicular failure.” [14].

For Topic II - age limits for fertility preservation, most countries (37 out of 44) introduced legal age limits in relation to public funding and access to ART, respectively, to fertility preservation. These limits applied to women in all cases, and six countries also set limits for men. Five countries had no age limits, and for two, no data was available. Some countries explicitly set a minimum age of 18 years for both men and women, such as Latvia, Lithuania, Montenegro, Ukraine [15], and the UK [16]. The highest maximum age limit for women was 55 years in Armenia, while the lowest maximum age limit was 45 years in Denmark, Belgium, Moldova, and France. Thus, the pre-menopausal and menopausal women are prevented from parenting. For men, age limits appeared in Austria (49 years), Ireland (59), Finland, France, Portugal (60), and Sweden (56). In Switzerland, the rule was that the potential parents should be able to be alive until the child is 18 years old [15].

Fourteen guidelines included age limits combined with clinical criteria for fertility preservation technologies. For example, according to FertiPROTEKT’s (network of German, Austrian, and Swiss institutions) recommendations, cryopreservation of ovarian tissue is recommended for younger women or children. The upper age limit is suggested at 35–38 years [17].

Regarding Topic III – type of preserved reproductive biomaterial, cryopreservation of gametes for medical conditions risking fertility was allowed in all European countries [15]. However, ovarian tissue preservation was permitted in only 25 countries. Four countries - Bulgaria, Croatia, Hungary, and Serbia - considered ovarian tissue cryopreservation experimental or had not implemented it.

Most guidelines (19 out of 26) included preservation of ovarian and/or testicular tissue alongside gamete preservation.

Posthumous usage (Topic IV) was allowed in 17 European countries and explicitly forbidden in 19. Data were missing for 8 countries. Explicit informed consent was required. In Belgium, both partners have to agree before death for posthumous embryo use. In the Czech Republic, although posthumous usage is not explicitly prohibited, written consent is required [18]. In Greece and Spain, men must give consent for posthumous use of their sperm. Some countries set time limits for postmortem IVF performance: Belgium and Greece - between 6 months and 2 years; Spain and North Macedonia - up to 12 months [19]; Ireland - not earlier than 12 months after the death [20]. No data was found on limits for how often posthumous material may be used.

Only 3 out of the studied guidelines addressed posthumous reproduction. They pointed to:

- Possible family disputes over gamete use after death [21].
- Patient information about legal rules on posthumous use [14].
- Recommendation for sperm cryopreservation before hazardous activities [22].

Informed consent (Topic V) was explicitly mentioned as a term in 23 of the studied law-informing documents. In 16 of them, the focus was on the right of the man or the woman to withdraw their consent for the use of preserved reproductive tissues or cells in assisted reproduction. 

Among the guidelines, 11 used the term “informed consent”. Two referred to “informed decision”, one to “shared decision-making”, and one to a “2-stage informed consent process”. Pre-treatment counselling was recommended in 10 guidelines (Additional file 3 in Supplementary Material), while 2 also highlighted the need for post-treatment counselling [3, 23].

For Topic VI - state/health insurance funding, strict funding rules for ART existed in all studied countries. These rules also applied to fertility preservation, since using cryopreserved material involves ART. In 41 out of 44 countries, some public funding supported ART procedures, medications, physician services, and laboratory tests. Three countries, Albania, Armenia, and Georgia, had no public funding for ART. Cryopreservation was funded, conditionally or unconditionally, in 16 countries. Except for France, no country funded non-medical egg or sperm freezing [24]. 

Funding was rarely mentioned in the guidelines. Only 9 guidelines referred to it, and without specific details. The ESMO (European Society for Medical Oncology) Clinical Practice Guideline called for universal insurance coverage for every cancer patient [25].

Storage periods (Topic VII) differed by country and type of reproductive material. In 11 countries, no explicit limit existed for some or all reproductive material. In 13 countries, data were missing. 

Seven guidelines mentioned storage but gave no time frames. They referred to national legal rules.

Social egg-freezing (Topic VIII) was allowed in 27 European countries. It was not allowed or not performed in 12 countries, i.e. Austria, Belarus, Bosnia and Herzegovina, the Czech Republic, Hungary, Lithuania, Luxembourg, Malta, Moldova, Poland, Serbia, and Slovenia. 

Only one guideline, the ESHRE (European Society of Human Reproduction and Embryology) Female Fertility Preservation Guideline [3], discussed social egg freezing. It appeared under “fertility preservation for age-related fertility loss”.

Regarding Topic IX - requirements towards partnership status, 32 countries allowed access to fertility preservation for single women. In 10 countries, only heterosexual couples had access. Two countries -Belgium and France - had no specific partnership requirements.

Eight guidelines mentioned partnership status. It appeared in the context of shared embryo ownership and risks of couples’ separation before embryo transfer [22, 25]. To guarantee woman’s independence, the FertiPROTEKT Practical Guideline [22] recommends storing 50% or 100% of oocytes unfertilized. 

Special provisions for transgender individuals (Topic X) existed in all 44 studied European countries. In 25 countries, cryopreservation of reproductive material of transgender individuals was allowed.

Six guidelines briefly addressed transgender individuals. ESHRE Female Fertility Preservation Guideline [3] emphasized care for transgender men and available cryopreservation options. The ESO-ESMO (European School of Oncology and European Society for Medical Oncology) guideline [26] called for more education and research to close counseling gaps. The FIGO (International Federation of Gynecology and Obstetrics) guideline [27] stressed multidisciplinary care for informed decisions. The British Fertility Society guideline [28] advised early fertility preservation for people transitioning from female to male, with counseling before storage and use. 

The 10 topics are further discussed through the prism of the four bioethical principles - autonomy, beneficence, non-maleficence, and justice [9] - to bring insights of ethical issues and their possible resolution. We found each topic related more strongly to some principles than others. These ethical links will be explored in the discussion.

## Discussion

### Autonomy

#### Definition

The principle of respect for autonomy means recognizing each person’s right to hold views, make choices, and act based on their own values and beliefs [9]. 

#### Related topics

Informed consent (Topic V); posthumous usage (Topic IV); social egg freezing (Topic VIII); requirements towards partnership status (Topic IX).

Informed consent (Topic V) is a classical representation of the principle of autonomy [9]. The laws required explicit written and renewed consent for ART, respectively, for fertility preservation. This applied both to living patients and posthumous reproduction. Consent had to remain valid until the procedure. However, the documents gave no detail on the content or process of informed consent. Therefore, the realisation of informed consent in clinical practice remains challenging, especially in the case of minors as patients. Recent views in support of respect for autonomy recommend that as children mature, they should be engaged in decisions about their care to respect their emerging autonomy and capacity to decide, rather than being excluded categorically [29]. Therefore, young people should be included in decision-making as much as possible. For example, the UK General Medical Council advises assessing whether a young person can understand and consent to the treatment [30]. Gametes may be stored without consent only in exceptional cases, in the child’s best interest, and must be destroyed after death [30].

Posthumous usage (Topic IV) also links to autonomy and supports this principle. The person's consent is needed for the use of their reproductive material after death [31]. This shows an extended view of autonomy where decisions made in life apply after death. Fulfilling legal requirements is essential. In the European Court of Human Rights' case Pejrilova v. the Czech Republic [32], the applicant's late husband never changed his mind or withdrew consent for assisted reproduction. Still, the postmortem procedure was denied because his consent was not given in the legally required form. No ethical arguments changed the court's ruling. 

Social egg freezing (Topic VIII) is closely related and supports the principle of respect for autonomy because it empowers women to control reproduction beyond biological age [33, 34], without needing donor oocytes [35]. Despite the fact that social egg freezing strengthens reproductive autonomy, studies show risks to free choice. These include lack of access to state/health insurance funding [36], i.e., lack of alternatives to funding and access only to women who have the financial means for the procedure and storage fees. Another risk to freedom of decision-making comes from the decisional pressure on the side of employers when the procedure is covered by the employer for the female employees in reproductive age [37].

The requirements for partnership status (Topic IX) affect autonomy by defining who can access fertility care. Therefore, these requirements oppose the autonomy principle. Where ART is allowed only for heterosexual couples, people outside this group cannot form families in the way they freely choose.

### Beneficence

#### Definition

The principle of beneficence means we have a moral duty to act for the benefit of others [9]. 

#### Related topics

Definitions of fertility preservation (Topic I); type of preserved reproductive material (Topic III); posthumous usage (Topic IV); requirements for partnership status (Topic IX).

Fertility preservation relates to the benefit of having one's own genetic children, thus supporting the principle of beneficence. That is why we studied definitions of fertility preservation and whether it is defined as a separate service (Topic I). The latter shows recognition of cryopreservation of reproductive biomaterial as separate from ART, meaning that all patients can access it, even without being included in fertility treatment through ART. Most documents, however, looked at cryopreservation of reproductive cells and tissues just as a step of ART and not as a separate service. This may limit access for people who could benefit from fertility preservation services without being subjected to fertility treatment through ART programmes. 

The type of preserved reproductive material (Topic III) supports beneficence by offering future chances for genetic parenthood [38]. When more types of materials are allowed for cryopreservation, more patients benefit. For example, ovarian or testicular tissue cryopreservation is the only option for fertility preservation for patients at prepubertal age. These methods were offered in many - but not all - countries. To address the issue of the unavailability of some fertility preservation technologies in certain countries, mechanisms for cross-border treatment and detailed informed consent, especially given the experimental nature of some fertility preservation technologies, should be developed. 

Posthumous usage (Topic IV) also relates to beneficence. In this case, the notion of beneficence is complex, i.e., it allows the woman to have a desired child even after the partner's death. However, the welfare of the childs stays highly debatable issue. Ethically problematic is the creation of a child who would be intentionally conceived as a half-orphan. This is a key consideration, for example, in the French law, where in cases of postmortem reproduction, the welfare of the future child is given priority over adult reproductive autonomy. An example here is the case of Baret and Caballero v. France [39] where both widows, Ms Baret and Ms Caballero, were not allowed to transfer cross-border cryopreserved sperm and embryos, despite the available explicit consent of their late husbands. Although an orphanage can happen in life, this was not a result of intentional medical action that makes the moral difference.

The genetic offspring, on the other side, can be considered benefit for the late partner. No laws set limits on how often this can happen. The focus is only on the time period after death. Perhaps, the underlying presumption was that the surviving partner would like to guarantee offspring once. However, precedents already exist and deserve reflection and further regulation efforts, if posthumous reproduction is allowed. The case of the British citizen Diane Blood drew attention in 1996. She used her late husband’s sperm to conceive two children in Belgium in 1998 and 2002. She later shared her legal battles in the book “Flesh and Blood” [40]. 

The requirements for the partnership status (Topic IX) oppose the principle of beneficence because they prevent single women from benefiting from ART, respectively fertility preservation.

### Non-maleficence

#### Definition

The principle of non-maleficence obligates us to avoid causing harm to others [9]. 

#### Related topics

Storage period (Topic VII); requirements for partnership status (Topic IX). 

The setting of a storage period (Topic VII) helps clinics manage unclaimed or unused gametes [41]. Thus, storage periods support the principle of non-maleficence. Limits on storage time also reflect classical non-maleficence concerns about medical risks of late pregnancies [42] and the social risks of late parenthood [43]. However, too short storage periods might fail to serve patients’ reproduction intention and make the fertility preservation useless [42].

The requirements for partnership status (Topic IX) oppose the principle of non-maleficence since they limit access to fertility preservation to heterosexual couples - traditional societal views of family [44]. These requirements aim to prevent reproduction outside this model. Risks like single parenthood [6] or stigmatization of children [43] are also seen as arguments in support of partnership requirements. Another reason is to avoid legal disputes over the ownership of the embryos after a couple’s separation. One example is the case of Evans v. the United Kingdom [31], where the applicant’s ex-partner withdrew consent to use their embryos. Though the embryos were applicant’s only chance to have children after cancer treatment, the European Court of Human Rights upheld the partner’s right to withdraw consent [31].

### Justice

#### Definition

The principle of justice requires equal treatment of all individuals [9], with a focus on equitable distribution amid limited resources, equal access to healthcare and non-discrimination in service provision [45]. 

#### Related topics

Age limits (Topic II); state/health insurance funding (Topic VI); social egg freezing (Topic VIII); storage period (Topic VII); requirements for partnership status (Topic IX); special provisions for transgender individuals (Topic X). 

Age limits (Topic II) raise justice concerns, as they may discriminate against older patients. Therefore, age limits oppose the principle of justice. While medical risks and societal norms may justify some limits [42, 46], setting them in law rather than through shared physician-patient decision-making, may restrict fair access. Most age restrictions apply only to women, raising additional concerns over gender equality [47].

State/health insurance funding (Topic VI) supports justice, as it ensures equal access. Although most countries provide some ART funding, few cover cryopreservation alone. Patients often share costs for medication, services, or tests, which - while sometimes necessary - can cause inequality in access. 

Funding also varies by reason: medical needs are often supported, while social egg freezing (Topic VIII) is typically self-funded. In France, employer coverage of social egg freezing is forbidden [24], but women are not disadvantaged as national health insurance covers the procedure. This is the first example showing that the reason for egg freezing is irrelevant when eligibility for funding is determined [48]. The mere terminology, in this case, is misleading as a criterion for funding decisions because not all medical reasons fall into “medical indications” for egg freezing according to the different laws, e.g., in Belgium since 2017 cancer patients, women with borderline ovarian tumours, and patients with hematopoietic disorders requiring stem cell transplantation can access public funding for egg freezing. However, women with primary ovarian insufficiency, endometriosis or Turner's syndrome are ineligible for public funding, despite these being conditions that many may consider as possessing the features that characterise a medical indication for egg freezing [48].

The specification of storage period (Topic VII) is another justice issue because it affects differently the user groups. For example, a healthy woman who has frozen her eggs in her early 30 s with a vision to guarantee a late pregnancy, cannot receive the benefits of the technology if the storage period is limited only to 5 or even 10 years [42]. 

Partnership status requirements (Topic IX) conflict with justice by denying access based on relationship type. Since justice calls for equal access, such criteria are ethically unjustified.

Some patient groups, such as transgender individuals (Topic X), were addressed separately in the law. As a newer group seeking fertility preservation, they have the same rights as others, such as cancer patients, for whom the technology was first developed. While laws in most countries were permissive, the range of services offered to transgender individuals remained narrower than for cisgender patients [49]. 

Additionally, fertility preservation for transgender individuals should be given proper consideration in clinical guidelines. Yet only four of the studied guidelines discussed transgender fertility preservation in detail [3, 22, 28, 49], and two mentioned it only briefly in general statements [26, 27].

**Fig. 2:**
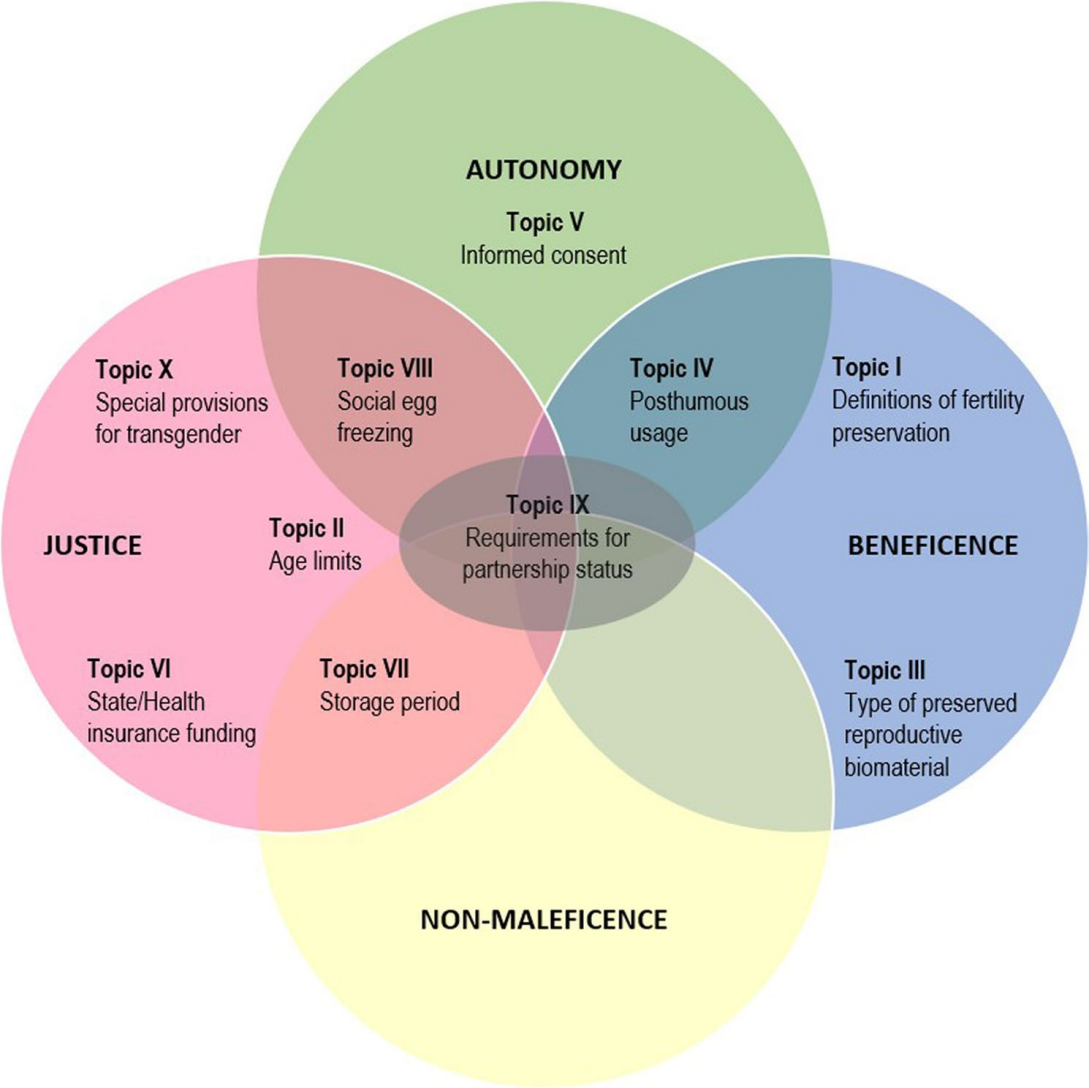
Correspondence of the studied topics to the four principles of bioethics

An overview of the discussed relations between the topics and the four principles of bioethics is presented in Fig. 2.

Among the studied topics, partnership status requirements, social egg freezing, and preservation of reproductive material for transgender individuals were the most strictly regulated. The European normative framework on fertility preservation thus seeks to balance two goals: maintaining reproduction within traditionally accepted age boundaries and ensuring equal access for all population groups (e.g., single women, married couples, transgender individuals). Regarding the latter, many countries still limit the reproduction of single women and transgender individuals. These limits touch upon the right to parent, which is not only a moral claim but also a component of reproductive rights frameworks [50]. It is a negative right requiring non-interference by others, especially the state. Individuals have a claim not to be unjustly prevented from becoming parents [50].

The ethical concerns raised in the discussion could be further addressed in clinical guidelines through the following improvements towards clearer communication:

- Topic I – Definitions of fertility preservation: Recognition of fertility preservation as separate from ART concept and service should be encouraged. Separate guidelines for fertility preservation, especially for non-oncological fertility preservation, should be adopted.
- Topic II - Age limits: Guidelines should explain how to ethically balance age with other factors when deciding on fertility preservation.
- Topic III – Types of preserved reproductive biomaterial: In order to address the unavailability of some fertility preservation options in some countries, guidelines should provide instructions on discussions of mechanisms for cross-border treatment. These should focus primarily on detailed informed consent but should not underestimate the experimental nature of certain fertility preservation techniques.
- Topic IV - Posthumous usage: Clear instructions should help physicians respond to posthumous reproduction requests, even in countries where such procedures currently are not allowed. Patients may still raise these questions out of ignorance or emotional need.
- Topic V - Informed consent: Guidelines should offer detailed advice on obtaining valid consent, especially in children and adolescents. Although national laws regulate this, fertility preservation poses unique challenges to be addressed also in the guidelines.
- Topic VI - State/health insurance funding: Guidance should be included on how to discuss complex funding schemes as part of the informed consent process.
- Topic VII - Storage periods: Clinical guidelines should offer clearer advice on how to communicate storage limits as part of general patient information.
- Topic VIII - Social egg freezing: Guidelines should include advice on protecting women’s autonomy and freedom of decision-making in decisions about social egg freezing.
- Topic IX - Requirements for partnership status: Clear instructions should help physicians respond to requests from single women, even in countries where they are deprived from access to fertility preservation services. Patients may still pose questions for transborder health care that results in different legal and ethical issues.
- Topic X - Special provisions for transgender individuals: Clinical guidelines should include specific directions for fertility preservation in transgender individuals, with clear attention to ethical considerations.

### Limitations

Our study has several limitations. First, the language barrier and lack of English versions of many laws prevented us from using them as primary sources. Second, the secondary sources we relied on were sometimes incomplete for our specific topics of interest and varied in publication date, meaning some legal changes may have occurred since. Third, in aiming to identify the most specific documents, we restricted our search terms, which may have led to the exclusion of some relevant documents. Fourth, for certain topics, we could not find relevant data for all countries in our study. Fifth, while fertility preservation overlaps with assisted reproductive technologies during the re-use of preserved biomaterial, the specifics of ART legislation fell outside our research scope. Sixth, our focus on officially published documents may not fully reflect actual clinical practice. Lastly, we aimed to provide a snapshot of the whole field of normative regulation of fertility preservation. Thus, we could not subject each studied topic to a more thorough ethical analysis.

Despite these limitations, we believe the body of information we used was sufficient to draw a comprehensive picture of the current European normative framework on fertility preservation and to support our ethical analysis - the main aim of this study.

## Conclusion

Fertility preservation is no longer a marginal topic. It is a growing field that connects advanced medical practice, legal regulation, and deeply personal experiences. Our research identified 10 major topics of interest in laws regulating fertility preservation. These same topics were also found in the guidelines. However, our expectation that they would be further developed and detailed there was not substantiated.

The ethical analysis, based on the four principles of bioethics, allowed us to highlight specific ethical challenges. It also enabled us to make concrete suggestions for improving the guidelines - particularly by adding ethical content related to the studied topics.

Legal development in this field demands specific expertise. It calls for collaboration between legal, medical, and ethical experts, and a higher level of managerial decision-making. This is why our focus was on identifying and ethically analysing common gaps and inconsistencies in the European normative framework rather than suggesting concrete legal changes.

Yet beyond laws and documents, at the heart of this field is the individual - someone facing uncertainty, hope, and difficult choices. We believe ethical reflection should not be separate from care. It should guide how fertility preservation is understood, offered, and supported.

We hope this article contributes to a more inclusive, ethically grounded, and legally coherent approach to fertility preservation - one that respects both the boundaries of law and the needs of people.

## Supplementary Information

Supplementary Material 1.

Supplementary Material 2.

Supplementary Material 3.

## Data Availability

All data generated or analysed during this study are included in this published article [and its supplementary information files].

## Abbreviations

ART: Assisted reproductive technologies
