# Supplementary material for: Ethical analysis of the European normative framework on fertility preservation
Source: BMC Med Ethics. 2026 Feb 2;27:38. doi: 10.1186/s12910-026-01393-8 (PMC12930747; doi:10.1186/s12910-026-01393-8)
Supplement: Supplementary file 1 — Supplementary Material 1. [file 12910_2026_1393_MOESM1_ESM.docx]

Additional file 1. Documents identified through the systematic literature search and included in the analysis

| No | Title | Year | Reference |
| --- | --- | --- | --- |
| Primary sources: laws (binding sources) | | | |
| 1 | REGULATION (EU) 2024/1938 OF THE EUROPEAN PARLIAMENT AND OF THE COUNCIL of 13 June 2024 on standards of quality and safety for substances of human origin intended for human application and repealing Directives 2002/98/EC and 2004/23/EC | 2024 | European Parliament, Council of the European Union. REGULATION (EU) 2024/1938 OF THE EUROPEAN PARLIAMENT AND OF THE COUNCIL of 13 June 2024 on standards of quality and safety for substances of human origin intended for human application and repealing Directives 2002/98/EC and 2004/23/EC. Official Journal of the European Union L series 17.7.2024. Available from: <http://data.europa.eu/eli/reg/2024/1938/oj> |
| 2 | Bulgarian Health Act | 2004 | Bulgarian Parliament. Bulgarian Health Act. Announcement, SG No. 70 of 10.08.2004, in force since 1.01.2005. (2023) <https://lex.bg/laws/ldoc%20/2135489147> [Accessed February 15, 2025] |
| 3 | Order H-2 on Assisted Reproduction Activities from 2 July 2023 | 2023 | Ministry of Health. Order No. H-2 of July 12, 2023 for assisted reproduction activities. (2023). <https://dv.parliament.bg/DVWeb/showMaterialDV.jsp;jsessionid=AA56A9BDF88EABB537C36EA841C41833?idMat=197485> [Accessed February 15, 2025] |
| 4 | Law n°2021-1017 of 2 August 2021 on bioethics | 2021 | LOI n° 2021-1017 du 2 août 2021 relative à la bioéthique. Légifrance - Journal officiel électronique authentifié n° 0178 du 03/08/2021 Available from: <https://www.legifrance.gouv.fr/jorf/id/JORFTEXT000043884384> |
| 5 | The Embryo Protection Act of 13 December 1990 | 1990 | Act on the Protection of Embryos. Federal Law Gazette, Part I, No. 69, issued in Bonn, 19th December 1990, page 2746 Available from: <https://www.bundesgesundheitsministerium.de/fileadmin/Dateien/3_Downloads/Gesetze_und_Verordnungen/GuV/E/ESchG_EN_Fassung_Stand_10Dez2014_01.pdf> |
| 6 | Health (Assisted Human Reproduction) Act No.18/2024 | 2024 | Health (Assisted Human Reproduction) Act No.18/2024. Available from: <https://www.irishstatutebook.ie/eli/2024/act/18/enacted/en/pdf> |
| 7 | Code of the Republic of Kazakhstan “On People’s Health and Healthcare System” No. 360-VI/July 7, 2020 | 2020 | Code of the Republic of Kazakhstan “On People’s Health and Healthcare System” No. 360-VI/July 7, 2020 Available from Adilet: Legal information system of Regulatory Legal Acts of Republic of Kazakhstan: <https://adilet.zan.kz/eng/docs/K2000000360> |
| 8 | Law No. 138 of 15.06.2012 regarding reproductive health | 2021 | Republic of Moldova Parliament. Law No. 138 of 15.06.2012 regarding reproductive health. Available from: <https://natlex.ilo.org/dyn/natlex2/natlex2/files/download/111901/MDA-111901%20(EN).pdf> |
| 9 | The Federal Law on Medically Assisted Reproduction 01.01.2001 amendment 01.09.2017 | 2001 | Federal Act on Medically Assisted Reproduction (Reproductive Medicine Act, RMA) of 18 December 1998 (Status as of 1 July 2023). Available from: <https://www.fedlex.admin.ch/eli/cc/2000/554/en> |
| 10 | Human Fertilisation and Embryology Act 1990, amended 2008 | 1990 | Parliament of the United Kingdom. Human Fertilisation and Embryology Act 1990. Available from: <https://www.legislation.gov.uk/ukpga/1990/37#commentary-key-3b876889c220097e5c8299fd22ab2954> |
| Secondary sources: law-informing articles | | | |
| 1 | Oocyte and ovarian tissue cryopreservation in European countries: statutory background,  practice, storage and use | 2017 | ESHRE Working Group on Oocyte Cryopreservation in Europe; Shenfield F, de Mouzon J, Scaravelli G, Kupka M, Ferraretti AP, Prados FJ, Goossens V. Oocyte and ovarian tissue cryopreservation in European countries: statutory background, practice, storage and use. Hum Reprod Open. 2017 Mar 29;2017(1):hox003. doi: 10.1093/hropen/hox003. |
| 2 | Between “Medical” and “Social” Egg Freezing A Comparative Analysis of Regulatory Frameworks in Austria, Germany, Israel, and the Netherlands | 2021 | Rimon-Zarfaty N, Kostenzer J, Sismuth LK, de Bont A. Between "Medical" and "Social" Egg Freezing : A Comparative Analysis of Regulatory Frameworks in Austria, Germany, Israel, and the Netherlands. J Bioeth Inq. 2021 Dec;18(4):683-699. doi: 10.1007/s11673-021-10133-z. |
| 3 | Replies by the member States to the questionnaire on access to medically assisted procreation (MAP), on the right to know about their origin for children born after MAP and on surrogacy 1 | 2023 | Council of Europe. Committee on Bioethics (DH-BIO). Replies by the member States to the questionnaire on access to medically assisted procreation (MAP), on the right to know about their origin for children born after MAP and on surrogacy. Strasbourg, 16 August 2023 Available from: <https://rm.coe.int/inf-2023-9-map-july-2023-replies-e/1680ad344f> |
| 4 | The Evolution of Legislation in the Field of Medically Assisted Reproduction and Embryo Stem Cell Research in European Union Members | 2014 | Busardò FP, Gulino M, Napoletano S, Zaami S, Frati P. The evolution of legislation in the field of Medically Assisted Reproduction and embryo stem cell research in European union members. Biomed Res Int. 2014;2014:307160. doi: 10.1155/2014/307160 |
| 5 | Survey on ART and IUI: legislation, regulation, funding and registries in European countries: The European IVF-monitoring Consortium (EIM) for the European Society of Human Reproduction and Embryology (ESHRE). | 2020 | Calhaz-Jorge C, De Geyter CH, Kupka MS, Wyns C, Mocanu E, Motrenko T, Scaravelli G, Smeenk J, Vidakovic S, Goossens V. Survey on ART and IUI: legislation, regulation, funding and registries in European countries: The European IVF-monitoring Consortium (EIM) for the European Society of Human Reproduction and Embryology (ESHRE). Hum Reprod Open. 2020 Feb 6;2020(1):hoz044. doi: 10.1093/hropen/hoz044 |
| 6 | Social freezing in Switzerland and worldwide – a blessing for women today? | 2013 | Wunder D. Social freezing in Switzerland and worldwide--a blessing for women today? Swiss Med Wkly. 2013 Feb 27;143:w13746. doi: 10.4414/smw.2013.13746. |
| 7 | Fertility Preservation for Non-Medical Reasons Controversial, but Increasingly Common | 2015 | von Wolff M, Germeyer A, Nawroth F. Fertility preservation for non-medical reasons: controversial, but increasingly common. Dtsch Arztebl Int. 2015 Jan 16;112(3):27-32. doi: 10.3238/arztebl.2015.0027. |
| 8 | Decision-making authority of patients and fertility specialists in Belgian law | 2007 | Penning G. Decision-making authority of patients and  fertility specialists in Belgian law. Reproductive BioMedicine Online. 2007, 15(1):19-23 |
| 9 | Fertility Preservation in Children and Adolescents during Oncological Treatment—A Review of Healthcare System Factors and Attitudes of Patients and Their Caregivers | 2023 | Pawłowski P, Ziętara KJ, Michalczyk J, Fryze M, Buchacz A, Zaucha-Prażmo A, Zawitkowska J, Torres A, Samardakiewicz M. Fertility Preservation in Children and Adolescents during Oncological Treatment-A Review of Healthcare System Factors and Attitudes of Patients and Their Caregivers. Cancers (Basel). 2023 Sep 2;15(17):4393. doi: 10.3390/cancers15174393. |
| 10 | The UK´s anomalous 10-year limit on oocyte storage: time to change the law | 2018 | Bowen-Simpkins P, Wang JJ, Ahuja KK. The UK´s anomalous 10-year limit on oocyte storage: time to change the law. Reprod Biomed Online. 2018 Oct;37(4):387-389. doi: 10.1016/j.rbmo.2018.07.004 |
| 11 | Fate, morals and rational calculations: Freezing eggs for non-medical reasons in Turkey. | 2018 | Kılıç A, Göçmen İ. Fate, morals and rational calculations: Freezing eggs for non-medical reasons in Turkey. Soc Sci Med. 2018 Apr;203:19-27. doi: 10.1016/j.socscimed.2018.03.014. |
| 12 | Social freezing of oocytes: a means to take control of your fertility. | 2020 | Wennberg AL. Social freezing of oocytes: a means to take control of your fertility. Ups J Med Sci. 2020 May;125(2):95-98. doi: 10.1080/03009734.2019.1707332. |
| 13 | Biomedical assisted fertilization in Macedonia, Serbia and Croatia ethical and legal aspects. | 2014 | Micković D, Ristov A. Biomedical assisted fertilization in Macedonia, Serbia and Croatia ethical and legal aspects. SEE Law Journal, 2014, 1(1):21-34. Available from: <https://www.seelawschool.org/pdf/1_Biomedical_Assisted_Fertilization_in_Macedonia_Serbia_and_Croatia_Ethical_and_Legal_Aspects.pdf> |
| 14 | Austrian Legislation and Jurisprudence on Medically Assisted Reproduction | 2021 | Balatinec I. Austrian Legislation and Jurisprudence on Medically Assisted Reproduction (2021). Available from: <https://medlawlab.web.auth.gr/wp-content/uploads/2021/12/Austrian-Legislation-and-Jurisprudence-on-Medically-Assisted-Reproduction.pdf> |
| 15 | Transgender persons' view on previous fertility decision-making and current infertility: a qualitative study. | 2024 | Asseler JD, de Nie I, van Rooij FB, Steensma TD, Mosterd D, Verhoeven MO, Goddijn M, Huirne JAF, van Mello NM. Transgender persons' view on previous fertility decision-making and current infertility: a qualitative study. Hum Reprod. 2024 Sep 1;39(9):2032-2042. doi: 10.1093/humrep/deae155. |
| 16 | Women’s viewpoints on egg freezing  in Austria: an online Q‑methodology study | 2021 | Kostenzer J, de Bont A, van Exel J. Women's viewpoints on egg freezing in Austria: an online Q-methodology study. BMC Med Ethics. 2021 Jan 6;22(1):4. doi: 10.1186/s12910-020-00571-6. |
| 17 | Conference consensus statement: Ethical and research dilemmas for fertility preservation in children treated for cancer | 2001 | Wallace WH, Walker DA. Conference consensus statement: ethical and research dilemmas for fertility preservation in children treated for cancer. Hum Fertil (Camb). 2001;4(2):69-76. doi: 10.1080/1464727012000199311. |
| 18 | Gender-affirming surgery for transgender Adolescents: Ethical and legal considerations | 2023 | Giordano S, Horowicz E. Gender-affirming surgery for transgender Adolescents: Ethical and legal considerations. Best Pract Res Clin Obstet Gynaecol. 2023 Feb;86:102295. doi: 10.1016/j.bpobgyn.2022.102295 |
| 19 | Mapping Accessibility to Fertility Preservation for Trans Masculine Individuals in The Netherlands | 2022 | Giacomozzi M, Aubin SG, Brancaccio MT. Mapping Accessibility to Fertility Preservation for Trans Masculine Individuals in The Netherlands. LGBT Health. 2022 Aug-Sep;9(6):369-383. doi: 10.1089/lgbt.2021.0302 |
| 20 | Eggs on Ice: Imaginaries of Eggs and  Cryopreservation in Denmark | 2018 | Rothmar Herrmann, J., & Kroløkke, C. (2018). Eggs on Ice: Imaginaries of Eggs and Cryopreservation in Denmark. NORA - Nordic Journal of Feminist and Gender Research, 26(1), 19–35. https://doi.org/10.1080/08038740.2018.1424727 |
| 21 | ESHRE. Comparative Analysis of Medically Assisted Reproduction in the EU: Regulation and Technologies (SANCO/2008/C6/051). Final Report. | 2008 | ESHRE. Comparative Analysis of Medically Assisted Reproduction in the EU: Regulation and Technologies (SANCO/2008/C6/051). Final Report. 2008. Grimbergen: ESHRE Central Office. p.166. Available from: <https://health.ec.europa.eu/document/download/3abc7b2a-cc86-4eac-8036-db18a34cb586_en> |
| 22 | Egg Cryopreservation for Social Reasons—A Literature Review | 2024 | Kynigopoulou S, Matsas A, Tsarna E, Christopoulou S, Panagopoulos P, Bakas P, Christopoulos P. Egg Cryopreservation for Social Reasons—A Literature Review. *Healthcare*. 2024; 12(23):2421. <https://doi.org/10.3390/healthcare12232421> |
| 23 | Fertility of tomorrow: Are there any restrictions left? | 2022 | Labrosse J, Grynberg M. Fertility of tomorrow: Are there any restrictions left? Ann Endocrinol (Paris). 2022 Jun;83(3):207-209. doi: 10.1016/j.ando.2022.04.011. |
| 24 | The revision of the French bioethics law and the questions it raises for the future of funding for egg freezing | 2022 | De Proost M, Johnston M. The revision of the French bioethics law and the questions it raises for the future of funding for egg freezing. Reprod Biomed Online. 2022 Apr;44(4):591-593. doi: 10.1016/j.rbmo.2021.12.002. |
| 25 | Assisted reproductive technology in France: The reproductive rights of LGBT people | 2023 | Agopiantz M, Dap M, Martin E, Meyer L, Urwicz A, Mougel R, Malmanche H. Assisted reproductive technology in France: The reproductive rights of LGBT people. J Gynecol Obstet Hum Reprod. 2023 Dec;52(10):102690. doi: 10.1016/j.jogoh.2023.102690. |
| 26 | The reproductive journeys of French women over 40 seeking assisted reproductive technology treatments in Spain | 2024 | Desy A, Marre D. The reproductive journeys of French women over 40 seeking assisted reproductive technology treatments in Spain. Soc Sci Med. 2024 Jun;351:116951. doi: 10.1016/j.socscimed.2024.116951. Epub 2024 May 8. PMID: 38743990. |
| 27 | National survey on the opinions of French specialists in assisted reproductive technologies about social issues impacting the future revision of the French Bioethics laws | 2020 | Creux H, Diaz M, Grynberg M, Papaxanthos-Roche A, Chansel-Debordeaux L, Jimenez C, Frantz S, Chevalier N, Takefman J, Hocké C. National survey on the opinions of French specialists in assisted reproductive technologies about social issues impacting the future revision of the French Bioethics laws. J Gynecol Obstet Hum Reprod. 2020 Nov;49(9):101902. doi: 10.1016/j.jogoh.2020.101902. |
| Primary sources: guidelines (non-binding sources) | | | |
| 1 | NICE “Fertility problems assessment and treatment” | 2017 | NICE. Fertility problems:assessment and treatment. Clinical guideline [CG156], Published:20 February 2013, Last updated: 06 September 2017. Available from: <https://www.nice.org.uk/guidance/cg156> |
| 2 | Standards of Care for the Health of Transgender and Gender Diverse People | 2022 | E. Coleman et al. (2022) Standards of Care for the Health of Transgender and Gender Diverse People, Version 8, International Journal of Transgender Health, 23:sup1, S1-S259, DOI:  10.1080/26895269.2022.2100644 |
| 3 | EUropean REcommendations for female FERtility preservation (EU-REFER): A joint collaboration between oncologists and fertility specialists | 2019 | Dolmans M et al. A. EUropean REcommendations for female FERtility preservation (EU-REFER): A joint collaboration between oncologists and fertility specialists. Crit Rev Oncol Hematol. 2019 Jun;138:233-240. doi: 10.1016/j.critrevonc.2019.03.010 |
| 4 | The Oncology Association of Bosnia and Herzegovina’s recommendations for fertility preservation in oncologic patients | 2022 | Cerić T et al. , The Oncology Association of Bosnia and Herzegovina's recommendations for fertility preservation in oncologic patients. Bosn J Basic Med Sci. 2022 Sep 16;22(5):646-650. doi: 10.17305/bjbms.2021.6977. |
| 5 | Fertility Preservation for Patients with Malignant Disease. Guideline of the DGGG, DGU and DGRM (S2k-Level, AWMF Registry No. 015/082, November 2017) – Recommendations and Statements for Girls and Women | 2017 | Dittrich R et al. Fertility Preservation for Patients with Malignant Disease. Guideline of the DGGG, DGU and DGRM (S2k-Level, AWMF Registry No. 015/082, November 2017) - Recommendations and Statements for Girls and Women. Geburtshilfe Frauenheilkd. 2018 Jun;78(6):567-584. doi: 10.1055/a-0611-5549. |
| 6 | ESGO/ESTRO/ESP Guidelines for the management of patients with endometrial carcinoma | 2021 | Concin N et al. ESGO/ESTRO/ESP guidelines for the management of patients with endometrial carcinoma. Int J Gynecol Cancer. 2021 Jan;31(1):12-39. doi: 10.1136/ijgc-2020-002230 |
| 7 | ESGO/ESHRE/ESGE Guidelines for the fertility-sparing treatment of patients with endometrial carcinoma | 2023 | Rodolakis A et al. ESGO/ESHRE/ESGE Guidelines for the fertility-sparing treatment of patients with endometrial carcinoma. Hum Reprod Open. 2023 Feb 6;2023(1):hoac057. doi: 10.1093/hropen/hoac057 |
| 8 | Female Fertility Preservation Guideline of the European Society of Human Reproduction and Embryology | 2020 | ESHRE Female Fertility Preservation Guideline Development Group. Female Fertility Preservation. Guideline of the European Society of Human Reproduction and Embryology, 2020. European Society of Human Reproduction and Embryology, Strombeek-Bever, Belgium, p.185. Accessible in Internet at: <https://www.eshre.eu/Guidelines-and-Legal/Guidelines/Female-fertility-preservation> |
| 9 | Fertility preservation and post-treatment pregnancies in post-pubertal cancer patients: ESMO Clinical Practice Guidelines | 2020 | Lambertini M et al. ESMO Guidelines Committee. Fertility preservation and post-treatment pregnancies in post-pubertal cancer patients: ESMO Clinical Practice Guidelines†. Ann Oncol. 2020 Dec;31(12):1664-1678. doi: 10.1016/j.annonc.2020.09.006 |
| 10 | ESO-ESMO fifth international consensus guidelines for breast cancer in young women (BCY5) | 2022 | Paluch-Shimon S et al. ESO-ESMO fifth international consensus guidelines for breast cancer in young women (BCY5). Ann Oncol. 2022 Nov;33(11):1097-1118. doi: 10.1016/j.annonc.2022.07.007 |
| 11 | ULAR recommendations for women's health and the management of family planning, assisted reproduction, pregnancy and menopause in patients with systemic lupus erythematosus and/or antiphospholipid syndrome | 2017 | Andreoli L et al. EULAR recommendations for women's health and the management of family planning, assisted reproduction, pregnancy and menopause in patients with systemic lupus erythematosus and/or antiphospholipid syndrome. Ann Rheum Dis. 2017 Mar;76(3):476-485. doi: 10.1136/annrheumdis-2016-209770 |
| 12 | Practical recommendations for fertility preservation in women by the FertiPROTEKT network. Part I: Indications for fertility preservation | 2018 | Schüring AN et al. Practical recommendations for fertility preservation in women by the FertiPROTEKT network. Part I: Indications for fertility preservation. Arch Gynecol Obstet. 2018 Jan;297(1):241-255. doi: 10.1007/s00404-017-4594-3 |
| 13 | Practical recommendations for fertility preservation in women by the FertiPROTEKT network. Part II: fertility preservation techniques | 2018 | von Wolff M et al. Practical recommendations for fertility preservation in women by the FertiPROTEKT network. Part II: fertility preservation techniques. Arch Gynecol Obstet. 2018 Jan;297(1):257-267. doi: 10.1007/s00404-017-4595-2 |
| 14 | Borderline ovarian tumors: French guidelines from the CNGOF. Part 2. Surgical management, follow-up, hormone replacement therapy, fertility management and preservation | 2021 | Bourdel N et al. Borderline ovarian tumors: French guidelines from the CNGOF. Part 2. Surgical management, follow-up, hormone replacement therapy, fertility management and preservation. J Gynecol Obstet Hum Reprod. 2021 Jan;50(1):101966. doi: 10.1016/j.jogoh.2020.101966 |
| 15 | Management of epithelial cancer of the ovary, fallopian tube, primary peritoneum. Long text of the joint French clinical practice guidelines issued by FRANCOGYN, CNGOF, SFOG, GINECO-ARCAGY, endorsed by INCa. (Part 2: systemic, intraperitoneal treatment, elderly patients, fertility preservation, follow-up) | 2019 | Lavoue V et al. Management of epithelial cancer of the ovary, fallopian tube, primary peritoneum. Long text of the joint French clinical practice guidelines issued by FRANCOGYN, CNGOF, SFOG, GINECO-ARCAGY, endorsed by INCa. (Part 2: systemic, intraperitoneal treatment, elderly patients, fertility preservation, follow-up). J Gynecol Obstet Hum Reprod. 2019 Jun;48(6):379-386. doi: 10.1016/j.jogoh.2019.03.018 |
| 16 | EMAS position statement: Fertility preservation | 2013 | Mintziori G et al. EMAS position statement: Fertility preservation. Maturitas. 2014 Jan;77(1):85-9. doi: 10.1016/j.maturitas.2013.10.010 |
| 17 | Fertility preservation for female patients with childhood, adolescent, and young adult cancer: recommendations from the PanCareLIFE Consortium and the International Late Effects of Childhood Cancer Guideline Harmonization Group | 2021 | Mulder RL et al. Fertility preservation for female patients with childhood, adolescent, and young adult cancer: recommendations from the PanCareLIFE Consortium and the International Late Effects of Childhood Cancer Guideline Harmonization Group. Lancet Oncol. 2021 Feb;22(2):e45-e56. doi: 10.1016/S1470-2045(20)30594-5. |
| 18 | Fertility preservation for male patients with childhood, adolescent, and young adult cancer: recommendations from the PanCareLIFE Consortium and the International Late Effects of Childhood Cancer Guideline Harmonization Group | 2021 | Mulder RL et al. Fertility preservation for male patients with childhood, adolescent, and young adult cancer: recommendations from the PanCareLIFE Consortium and the International Late Effects of Childhood Cancer Guideline Harmonization Group. Lancet Oncol. 2021 Feb;22(2):e57-e67. doi: 10.1016/S1470-2045(20)30582-9 |
| 19 | Communication and ethical considerations for fertility preservation for patients with childhood, adolescent, and young adult cancer: recommendations from the PanCareLIFE Consortium and the International Late Effects of Childhood Cancer Guideline Harmonization Group | 2021 | Mulder RL et al. Communication and ethical considerations for fertility preservation for patients with childhood, adolescent, and young adult cancer: recommendations from the PanCareLIFE Consortium and the International Late Effects of Childhood Cancer Guideline Harmonization Group. Lancet Oncol. 2021 Feb;22(2):e68-e80. doi: 10.1016/S1470-2045(20)30595-7 |
| 20 | Multidisciplinary consensus on the criteria for fertility preservation in cancer patients | 2021 | Santaballa A et al. Multidisciplinary consensus on the criteria for fertility preservation in cancer patients. Clin Transl Oncol. 2022 Feb;24(2):227-243. doi: 10.1007/s12094-021-02699-2. |
| 21 | SEOM Clinical Guideline of fertility preservation and reproduction in cancer patients | 2016 | Muñoz M et al. SEOM Clinical Guideline of fertility preservation and reproduction in cancer patients (2016). Clin Transl Oncol. 2016 Dec;18(12):1229-1236. doi: 10.1007/s12094-016-1587-9. |
| 22 | Fertility Preservation in Oncological and Non-Oncological Diseases. A Practical Guide. | 2020 | von Wolff M., Nawroth F. Fertility preservation in Oncological and Non-Oncological DIseases. A Practical Guide. 2020. Sham, Switzerland: Springer |
| 23 | Management of endometriosis: CNGOF/HAS clinical practice guidelines | 2018 | Collinet P et al. Management of endometriosis: CNGOF/HAS clinical practice guidelines - Short version. J Gynecol Obstet Hum Reprod. 2018 Sep;47(7):265-274. doi: 10.1016/j.jogoh.2018.06.003 |
| 24 | FIGO statement: Fertility preservation | 2023 | Henry L et al. FIGO statement: Fertility preservation. Int J Gynaecol Obstet. 2023 Dec;163(3):790-794. doi: 10.1002/ijgo.15187. |
| 25 | Management of Germ Cell Tumours of the Testis in Adult Patients. German Clinical Practice Guideline. Part I: Epidemiology, Classification, Diagnosis, Prognosis, Fertility Preservation, and Treatment Recommendations for Localized Stages | 2021 | Kliesch S et al. Management of Germ Cell Tumours of the Testis in Adult Patients. German Clinical Practice Guideline Part I: Epidemiology, Classification, Diagnosis, Prognosis, Fertility Preservation, and Treatment Recommendations for Localized Stages. Urol Int. 2021;105(3-4):169-180. doi: 10.1159/000510407. |
| 26 | Fertility preservation for medical reasons in girls and women: British fertility society policy and practice guideline | 2018 | Yasmin E et al. British Fertility Society. Fertility preservation for medical reasons in girls and women: British fertility society policy and practice guideline. Hum Fertil (Camb). 2018 Apr;21(1):3-26. doi: 10.1080/14647273.2017.1422297 |
