# Supplementary material for: Ethical analysis of the European normative framework on fertility preservation
Source: BMC Med Ethics. 2026 Feb 2;27:38. doi: 10.1186/s12910-026-01393-8 (PMC12930747; doi:10.1186/s12910-026-01393-8)
Supplement: Supplementary file 2 — Supplementary Material 2. [file 12910_2026_1393_MOESM2_ESM.docx]

Additional file 2. Overview of results of thematic analysis of laws regulating fertility preservation by country

| Country | Law involved with fertility preservation | Source | Topic I –  Definitions of fertility preservation | Topic II -  Age limits for fertility preservation/ ART access | Topic III –  Type of preserved reproductive biomaterial | Topic IV –  Posthumous usage | Topic V –  Informed consent | Topic VI –  State/Health insurance funding | Topic VII –  Storage period | Topic VIII –  Social egg freezing | Topic IX –  Requirements for partnership status | Topic X –  Special provisions for transgender individuals |
| --- | --- | --- | --- | --- | --- | --- | --- | --- | --- | --- | --- | --- |
| 1. Albania | No | Secondary^1^ | No data | Women 50 years | Allowed for medical reasons | No data | No data | No public funding at all^1^ | No data | ART not possible for single women | Heterosexual couples | No. Gender reassignment is not allowed^1^ |
| 1. Armenia | Law of the Republic of Armenia of December 26, 2002, no. Zr-474 (as amended on 05-08-2024) | Secondary^1^ | No data | 55 years | Allowed for medical reasons | No data | No data | No public funding at all^1^ | No data | No data | Heterosexual couples and Single women | No. Gender reassignment is not allowed^1^ |
| 1. Austria | The Reproductive Medicine Act 1992 | Secondary^1,2,3,5,29^ | Yes | Women 39 years  Men 49 years | Oocytes, embryos, ovarian tissue for cancer patients and benign diseases.^29^ | Not allowed. | - Explicit consent not older than 2 years.^3^  - The man’s consent can be revoked only up to the point of fertilization.^5^ | - 70% of medication and doctor’s costs.^1^  - Partial funding for medical egg freezing on case-by-case basis depending on the insurer^2^. | 10 years | Not allowed | Married or stable couples; since 2015 also lesbian couples. | Yes. ART with previously cryopreserved gametes is allowed.^1^ |
| 1. Belarus | Law of the Republic of Belarus of January 7, 2012 no. 341-z about assisted reproductive technologies (as amended on 28-12-2023) | Secondary^1^ | No data | Women 50 years | Allowed for medical reasons | No data | No data | Medication is fully covered but doctor costs vary per center.^1^ | No data | Not allowed | Heterosexual couples and Single women^1^ | No. Gender reassignment is not allowed^1^ |
| 1. Belgium | Medically Assisted Procreation and Destination of Surplus Embryos and Gametes Act of 06-07-2007^9^ | Secondary^1,4,5^ | No data | - 45 years for oocytes retrieval;  - 47 years for embryo transfer;  - no age limit for men | Oocytes, embryos, ovarian tissue for cancer patients and benign diseases.^29^ | - Possible for embryos with the explicit consent of both partners.  -6 months to 2 years after death. | Explicit and can be withdrawn at any stage up to the moment of embryo implantation^5^ | Oocytes cryopreservation for medical reasons. Age limit for women for ART funding 42 years. | 10 years; exception for minors | Allowed | No requirement | Yes. ART with previously cryopreserved gametes is allowed.^1^ |
| 1. Bosnia and Herzegovina | Health Insurance Legislation bylaws^9^ | Secondary^1,9^ | No data | Women 42 years | Allowed for medical reasons | No data | No data | Only private ART centers exist but patients can get public funding for the treatment; 50% of medication costs.^1^ | No data | Not performed | Heterosexual couples^1^ | No. Gender reassignment is not allowed^1^ |
| 1. Bulgaria | Bulgarian Health Act and Order H-2 on Assisted Reproduction Activities from 2 July 2023 | Primary^6,7^  Secondary^1,29^ | No | Women 51 years | - Oocytes and embryos for cancer patients and benign diseases.^29^  - Cryopreservation of ovarian tissue is experimental.^29^ | No. | -Required in written form.  -Despite its availability, gametes and embryos are destroyed after person’s death. | Fully covered ART till 43 years of age of the woman. | Defined by the patients but by recommendation should not exceed 5 years | Possible but not funded | Heterosexual and female couples and Single women^1^ | Yes. ART with previously cryopreserved gametes is allowed.^1^ |
| 1. Croatia | Law on medically assisted reproduction 2012^9^ | Secondary^1,8,10,29^ | No data | - 42 years for usage of oocytes;  -No age limit for embryos.^29^ | - Oocytes, embryos for cancer patients and benign diseases^29.^  - Cryopreservation of ovarian tissue is not implemented^29^ | Not allowed^10^ | Yes, written consent is required | Cryopreservation of oocytes and embryos is covered.^29^ | 5 years, can be prolonged with a written consent | Possible but not funded | Heterosexual couples and Single women^1^ | Yes. Oocyte cryopreservation is allowed^29^ |
| 1. Cyprus | Medically Assisted Human Reproduction Law 69(I)/2015 | Secondary^1,17,29^ | No data | Women 50 years | Allowed for medical reasons | Permitted^17^ | No data | - Only private ART centers exist but patients can get public funding for the treatment up to a specific amount.^1^  - Cryopreservation of oocytes, embryos and ovarian tissue are not covered.^29^ | 10 years for oocytes, embryos and ovarian tissue; can be prolonged^29^ | Possible | Heterosexual couples and Single women^1^ | Yes. Cryopreservation of oocytes, embryos, and ovarian tissue is allowed^29^. |
| 1. Czech Republic | Czech Act on Specific Health Services (Act No.373/2011)^9^ | Secondary^1,8,9,10,29^ | No data | Women 49 years | Oocytes, embryos, ovarian for cancer patients, but not for benign diseases.^29^ | No explicit prohibition but explicit written informed consent^10^ | Written consent should be renewed before each attempt for ART^10^ | Available both in private and public centers for women till 49 years; covered to a specified limit.^1^ | No limit^29^ | Not allowed^29^ | Heterosexual couples^1^ | No. Cryopreservation of oocytes, embryos, and ovarian tissue is not allowed^29^. |
| 1. Denmark | Act on medically assisted procreation in connection with medical treatment, diagnosis and research (Loi no.460/1997)^9^ | Secondary^1,8,9,10,29^ | No data | - 35 years for usage of oocytes;  - 45 years for embryos^29^ | Cryopreservation of oocytes, embryos, ovarian tissue are allowed for cancer patients and benign diseases.^29^ | Allowed^10^ | The man or the woman can freely withdraw consent at any stage up to embryo’s implantation^5.^ | - Yes, provided that the woman has no children and the woman is up to 40 years.  - Medication is covered to a specified maximum.^1^ | 5 years | Allowed in licensed centers | Heterosexual and female couples and Single women^1^ | Yes. Cryopreservation of oocytes, embryos, and ovarian tissue is allowed^29^. |
| 1. Estonia | Artificial Insemination and Embryo Protection Act 1997, amended 2018^9^ | Secondary^1,5,8,9,10^ | No data | Women 50 | Allowed for medical reasons | Allowed^10^ | The man’s consent can be revoked only up to the point of fertilization; after that the woman decides alone.^5^ | Yes, for women up to 40 | For embryos up to 7 years | Allowed | Heterosexual and female couples and Single women^1^ | No. Gender reassignment is not allowed^1^ |
| 1. Finland | Act on Assisted Fertility Treatments 1237/2006^9^ | Secondary^1,8,9,29^ | No data | Women 40-45 years  Men 60 years (not in the law but in practice)^1^ | Oocytes, embryos, ovarian tissue for cancer patients and benign diseases.^29^ | Not allowed.^8^ | In practice the man or the woman can freely withdraw consent at any stage up to embryo’s implantation^5^ | - Till 40-45 year;  - 65% of medication and some of the doctors' costs is covered.^1^ | Defined by the clinic^29^ | Allowed | Heterosexual and female couples and Single women^1^ | Yes. ART with previously cryopreserved gametes is allowed.^1^ |
| 1. France | Law n°2021-1017 of 2 August 2021 on bioethics | Primary^11^  Secondary^1,5,12,13^ | Yes | Re-usage of frozen material for women until 45 years and for men until 60 years of age | - Oocytes, embryos, ovarian tissue for cancer patients and benign diseases.^29^  - Oocyte and sperm cryopreservation for everyone who wishes; 29 to 37 years for women and 29 to 45 - for men. | - Not allowed. | - Consent is renewed each year (Art.31.III).  - For minors reaching the majority personal information about the conditions of storage is provided. (Art.31.I)  - The man/ woman can freely withdraw consent at any stage up to embryo’s implantation^5^ | - Fertility preservation procedures are completely reimbursed with a limit of IVF cycles^14^ and age limit for women 43.  - Partial reimbursement of the cost for "non-medical" egg freezing.^15^ | - In the absence of a response from the adult for ten consecutive years, the conservation of his or her gametes or germinal tissues is terminated. | Allowed | No requirement | Yes. ART with previously cryopreserved gametes is allowed.^1^ |
| 1. Georgia | The law of Georgia on Health Care 1997 - Chapter XXIII Family Planning^9^ | Secondary^1,9,29^ | No data | The age limit for use of stored oocytes, embryos or ovarian tissue is not regulated^29^ | Oocytes, embryos, ovarian tissue for cancer patients and benign diseases.^29^ | No data | No data | No public funding at all ^1^ | No data | Allowed^29^ | Heterosexual couples and Single women^1^ | No. Gender reassignment is not allowed^1^ |
| 1. Germany | The Embryo Protection Act of 13 December 1990 | Primary^16^  Secondary^1,5,29^ | No | The maximum recommended age for usage of stored oocytes is 50 years^29^ | Oocytes, embryos, ovarian tissue for cancer patients and benign diseases.^29^ | Not allowed^10^ | In practice the man or the woman can freely withdraw consent at any stage up to embryo’s implantation^5^ | - Medical egg freezing is covered including medication and preservation.^1^  - Age limit of ART funding for women between 25 and 39 years and for men between 25 and 49.^1^ | Defined by clinic^29^ | Possible | Heterosexual and female couples and Single women^1^ | Yes. Cryopreservation of oocytes, embryos, and ovarian tissue is allowed^29^. |
| 1. Greece | Law on Medical assistance on human assisted procreation 2002 and  Law on Application of medically assisted procreation 2005^9^ | Secondary^1,5,8,9^ | No data | Women 50 years | Allowed for medical reasons | - Allowed^10^  - 6 months to 2 years of man’s death^8^  - The man should give explicit written consent. | The man or the woman can freely withdraw consent at any stage up to embryo’s implantation.^5^ | Medications and laboratory tests are covered, except PGT, but medical services should be paid by the patients.^1^ | 5 years and can be extended till the woman reaches 50 years; maximum extension up to 20 years | Possible | Heterosexual couples and Single women^1^ | Preservation of gametes of transgender individuals is not allowed.^1^ |
| 1. Hungary | Health Act, Chapter IX, 2007 and  Ministerial Decree 30/1998^9^ | Secondary^1,8,9,29^ | No data | Women 49 years | - Oocytes and embryos for cancer patients and benign diseases.^29^  - Ovarian tissue cryopreservation is experimental.^29^ | Not allowed^8^ | If no written objection of the man exists, the woman has the right to decide to use the preserved embryos.^5^ | - ART medication is 90% covered. Doctors' costs are fully covered.^1^  - Women till 45 years.  - The storage fees are not covered. | - 10 years for oocytes and embryos;  - no limit for ovarian tissue^29^ | Not allowed | Heterosexual couples and single women. Lesbians are considered single women.^1^ | Preservation of gametes of transgender individuals is not allowed.^1^ |
| 1. Iceland | Artificial Fertilisation Act No.55/1996^9^ | Secondary^1^ | No data | No data | Oocytes, embryos, ovarian tissue for cancer patients and benign diseases.^29^ | No data | The man can withdraw consent at any stage up to the embryo’s implantation.^5^ | - Limits on how many times IVF is performed.  - No age limits.  - Cryopreservation of oocytes and embryos for cancer patients is covered; for benign diseases is only partially reimbursed.^29^ | No data | No data | Heterosexual and female couples and Single women^1^ | Yes. ART with previously cryopreserved gametes is allowed.^1^ |
| 1. Ireland | Health (Assisted Human Reproduction) Act 2024 | Primary^19^  Secondary^1,29^ | Yes. ART is recognised as treatment necessary to enable fertility preservation (Part 1,16) | Women 40 years  Men 59 years | Oocytes and ovarian tissue for cancer patients and benign diseases.^29^ | Permitted but cannot be performed earlier than 12 months after the death^19^ | Yes. Can be revoked. | - Medication up to a limit.  - Cryopreservation of oocytes and ovarian tissue for cancer patients is covered; for benign diseases, reimbursement is under conditions.^29^ | The storer can dispose of the storage after 10 years. Extension is possible (Part 4,40). | Allowed | Heterosexual and female couples and Single women^1^ | Yes. ART with previously cryopreserved gametes is allowed.^1^ |
| 1. Italy | Rules Concerning Medical Assisted Reproduction No 40/2004^9^ | Secondary^1,8,9^ | No data | - Cryopreservation of male and female gametes with written consent^9^  ^-^ Women 46 - 50 years in some regions. | Allowed for medical reasons | Allowed^10^ | The man’s consent can be revoked only up to the point of fertilization; after that the woman decides alone.^5^ | - Covers ART except for PGT and patients pay costs depending on the Regional local Health authority.  - Age limit for women 46.^1^ | No data | Allowed | Heterosexual couples^1^ | Yes. Cryopreservation of oocytes and ovarian tissue is allowed^29^. |
| 1. Kazakhstan | Code of the Republic Kazakhstan “On People’s Health and Healthcare systems” No.360-VI/2020^20^ | Primary^20^  Secondary^1^ | No | - Minimal age limit for women 19 years  - No maximum age limit | Allowed for medical reasons | No data | Required | ART is covered; no patients contributions^1^ | No data | No data | Heterosexual couples and single women^1^ | No. Gender reassignment is not allowed^1^ |
| 1. Latvia | Law on reproductive and sexual Health 2002^9^ | Secondary^1,8^ | No data | Minimal age limit 18 years for women and men | Allowed for medical reasons | Possible with explicit consent^8^ | No data | - Only private ART centers exist but patients can get public funding for the treatment.^1^  - Age limit for women 38.^1^ | Gametes stored for more than 10 years can be destroyed^8^ | Allowed | Heterosexual and lesbian couples and Single women^1^ | Yes. ART with previously cryopreserved gametes is allowed.^1^ |
| 1. Lithuania | The Law on the Assisted Reproduction of the Republic of Lithuania 14 September 2016 | Secondary^1,17,29^ | No data | - Minimal age limit 18 years for women and men.  - No age limit for the usage of material^29^ | Oocytes, embryos, ovarian tissue for cancer patients and benign diseases.^29^ | Allowed^17^ | No data | - Funding for medication, and up to limits for doctor’s costs. Age limit for women 42.  - Cryopreservation is not funded.^1^ | Embryos are stored up to 10 years after ART | Not allowed | Heterosexual couples^1^ | No. Gender reassignment is not allowed^1^ |
| 1. Luxembourg | No | Secondary^8,9^ | No data | Women 43 | No data | Not allowed^8^ | No data | Ovarian stimulation and ART within age limit | No data | Not allowed | No data | No data |
| 1. Malta | Embryo Protection Act 2018 | Secondary^1,17,18^ | No data | Women 48 | Allowed for medical reasons | Allowed^17^ | No data | - No for couples or women with child(ren).  - Medication is not covered. Doctor’s costs and test are covered for women till 48 years. | No data | Not allowed^18^ | Heterosexual and lesbian couples and Single women^1^ | Yes. ART with previously cryopreserved gametes is allowed.^1^ |
| 1. Moldova | Law No. 138 of 15.06.2012 regarding reproductive health | Primary^21^  Secondary^1^ | - Yes.  - Art.2. Fertility preservation - cryopreservation of reproductive tissues or cells to preserve their reproductive capacity. The process of saving or protecting a person's oocytes, sperm, and/or reproductive tissue (ovarian tissue, testicular tissue) so that they can use them to try to have biological children later in life.^21^ | - Up to 45 years with one’s own oocytes;  - up to 50 years with donor’s ova | Allowed for medical reasons | - Not allowed. | - The use of ART is possible only on the basis of written informed consent, valid for a single procedure (Art.9,6).  - Consent can be revoked until the moment of insemination or transfer of the embryo into the woman’s body (Art.9,9). | - ART fully covered.  - Age limit for women 40 years. | No data | Not performed | Heterosexual couples and Single women^1^ | No. Gender reassignment is not allowed^1^ |
| 1. Montenegro | Law on Medically Assisted Reproduction | Secondary^1,29^ | No data | - Minimal age limit 18 years for women and men^1^.  - 48 years for the usage of stored oocytes;  - 45-47 years for embryos;  - The usage of ovarian tissue is not regulated^29^ | Oocytes, embryos, ovarian tissue for cancer patients and benign diseases.^29^ | No data | No data | - ART covered except for some medication.  - Women till 44 years.  - Cryopreservation of oocytes, embryos and ovarian tissue is not covered.^29^ | Defined by clinic, current practice is 2 times for 3 to 5 years^29^ | Allowed | Heterosexual couples and Single women^1^ | Yes. Cryopreservation of oocytes, embryos, and ovarian tissue is allowed^29^. |
| 1. Netherlands | Embryos Law 20.06.2002 | Secondary^1,5,26,29^ | No data | 49 years for usage of stored oocytes, embryos and ovarian tissue. | Oocytes, embryos, ovarian tissue for cancer patients and benign diseases.^29^ | Allowed^10^ | The man or the woman can freely withdraw consent at any stage up to embryo’s implantation.^5^ | - Yes.  - Fertility preservation procedures including cryopreservation;  - Women till 42 years.^1^ | No limit^29^ | Allowed | Heterosexual and lesbian couples and Single women^1^ | Fertility preservation for transgender is covered^26^ |
| 1. North Macedonia | Law on Biomedical Assisted Fertilization No. 37/2008 | Secondary^1,22^ | No data | No data | Allowed for medical reasons | - Allowed.  - Within 1 year after the death with a signed consent (Art.33,2).^22^ | No data | Coverage of medication up to a limit.^1^ | No data | No data | Heterosexual couples and Single women^1^ | No. Gender reassignment is not allowed^1^ |
| 1. Norway | The act relating to the application of biotechnology in human medicine 2003 | Secondary^1,8,9,10,29^ | No data | 46 years for usage of stored oocytes, embryos and ovarian tissue | Oocytes, embryos, ovarian tissue for cancer patients and benign diseases.^29^ | Not allowed^10^ | No data | - Cryopreservation of oocytes, embryos and ovarian tissue is covered.^29^  - ART covered up to a limited number of cycles ; differences across the country.^8^ | - Not defined for oocytes, extending natural fertility is not allowed;  - 5 years for embryos;  - no limit for ovarian tissue^29^ | Allowed^29^ | Heterosexual and lesbian couples and Single women^1^ | Yes. Cryopreservation of oocytes is allowed^29^. |
| 1. Poland | Poland’s Medical Profession Act of 1996^8^ | Secondary^1,8,17,29^ | No data | The age limit for usage of stored oocytes, embryos and ovarian tissue is not defined. | Oocytes, embryos, ovarian tissue for cancer patients and benign diseases.^29^ | Allowed^17^ | No data | - Medication for ART.  - No for cryopreservation.^29^  - Women till 40 years.^1^ | - No limit for oocytes and ovarian tissue,  - 20 years for embryos^29^ | Not allowed^29^ | Heterosexual couples^1^ | Transgender have no access to ART. |
| 1. Portugal | Law No. 17/2016^9^ | Secondary^1,8,17^ | No data | Women 50 years  Men 60 years | Oocytes and ovarian tissue for cancer patients and benign diseases.^29^ | Not allowed^8,17^ | No data | - Doctor’s costs, laboratory tests fully funded; medication up to a limit.  - Women till 40 years and men til 60.  - Cryopreservation of oocytes and ovarian tissue.^29^ | 5 years and can be extended | Allowed | Heterosexual and lesbian couples and Single women^1^ | Yes. ART with previously cryopreserved gametes is allowed.^1^ |
| 1. Romania | Law 63/2012^8^ | Secondary^1,8,29^ | No data | - 48 years with own oocytes;  - 50 years with donor’s ova | Oocytes, embryos, ovarian tissue for cancer patients and benign diseases.^29^ | Not allowed^8^ | No data | - Limits for female BMI, age till 40 years, and availability of child(ren).  - Medication^1^ and cryopreservation are not covered.^29^ | No limit^29^ | Allowed | Heterosexual and lesbian couples and Single women^1^ | Yes. Cryopreservation of oocytes, embryos, and ovarian tissue is allowed^29^. |
| 1. Russian Federation | The Federal Law “On Fundamentals on Protection of Citizens Health” 2011 No. 323-FZ | Secondary^1,29^ | No data | No limit for stored oocytes, embryos and ovarian tissue^29^ | Oocytes, embryos, ovarian tissue for cancer patients and benign diseases.^29^ | Not allowed | No data | - ART is covered.  - Cryopreservation is not funded; under conditions only for embryos.^29^ | No limit^29^ | Allowed | Heterosexual couples and Single women.^1^ | Yes. ART with previously cryopreserved gametes is allowed.^1^ |
| 1. Serbia | Act on the Treatment of Infertility with Biomedically Assisted Fertilization Procedures 2017 | Secondary^1,8,22,29^ | No data | Ban of access to ART for older women^22^ | - Oocytes and embryos for cancer patients and benign diseases.  - Ovarian tissue only for cancer patients.^29^ | Not allowed^8^ | No data | - Limit for maximum female BMI.  - Cryopreservation of oocytes and embryos is covered.^29^ | No limit for oocytes, 5 years for embryos; can be extended^29^ | Not allowed | Heterosexual couples and Single women^1^ | No. Gender reassignment is not allowed^1^ |
| 1. Slovakia | Law 277/1994 on Health Care^8^ | Secondary^1,8,10^ | No data | Women 50 years | Allowed for medical reasons | Not allowed^10^ | No data | - ART is funded.  - Medications up to a limit.^1^ | No data | Allowed | Heterosexual couples^1^ | No. Gender reassignment is not allowed^1^ |
| 1. Slovenia | Law on Biomedically Assisted Fertilisation No. 70/2000^8^ | Secondary^1,8,10,29^ | No data | Reproductive age limit for women^29^ | Oocytes, embryos, ovarian tissue for cancer patients and benign diseases.^29^ | Not allowed^10^ | No data | - Cryopreservation of oocytes and embryos.^1^  - Cryopreservation of ovarian tissue is covered.^29^  - Women up to 42 years. | 10 years for oocytes, embryos and ovarian tissue^29^ | Not allowed | Heterosexual couples^1^ | Previous gamete and gonadal tissue is not allowed for transgender^1^ |
| 1. Spain | Law on Assisted Human Reproduction Techniques No.14/2006 | Secondary^1,5,8, 29^ | No data | 50 years for usage of stored oocytes, embryos or ovarian tissue in practice.^29^ | Oocytes, embryos, ovarian tissue for cancer patients and benign diseases.^29^ | Allowed up to 12 months after man’s death with explicit consent^8^ | Man’s right to revoke consent is valid when he is married and lives with the woman.^5^ | - Cryopreservation is covered.^29^  - Limit on female BMI.  - Women till 40 years and men till 55 years. | No limit^29^ | Allowed | Heterosexual and lesbian couples and Single women^1^ | Yes. ART with previously cryopreserved gametes is allowed.^1^ |
| 1. Sweden | Genetic Integrity Act No. 351/18.05.2006 | Secondary^1,5,8,23.24,29^ | No data | Men 56 by recommendation^1^  Women 45-50 years for usage of oocytes, embryos and ovarian tissue.^29^ | Oocytes, embryos, ovarian tissue for cancer patients and benign diseases.^29^ | Not allowed^8^ | The man can withdraw consent at any stage up to embryo’s implantation.^5^ In case of children consent is required from parents and the patient.^23^ | - Limit on female BMI  - Women till 39 and men till 55.  - Medication and doctor’s costs up to a limit.^1^  - Cryopreservation is covered.^29^ | No time limits for the storage of sperm and oocytes | Possible, no specific law^24^ | Heterosexual and lesbian couples and Single women^1^ | Yes. ART with previously cryopreserved gametes is allowed.^1^ |
| 1. Switzerland | The Federal Law on Medically Assisted Reproduction 01.01.2001 amendment 01.09.2017 | Primary^25^  Secondary^1,2,10,29^ | Yes. | - Only married couples may use donated sperm cells (Art.3,3).  - No age limit for the usage of stored oocytes, embryos or ovarian tissue^29^ | Oocytes, embryos, ovarian tissue are allowed for cancer patients and benign diseases.^29^ | - Not allowed^10^  - Reproductive cells or impregnated ova may not be used after the death of the person from whom they were obtained.^25^ | - The man or the woman can freely withdraw consent at any stage up to embryo’s implantation.^5^  - The couple’s written consent is required for the reactivation of preserved embryos and impregnated ova.^25^ | - Cryopreservation of mature oocytes and ovarian tissue are until the 40^th^ birthday when the risk of amenorrhea induced by the gonadotoxic treatment is >20%.^29^ | - 5 years; can be extended once by another 5 years.  - Longer storage for medical indications.  - For embryos 10 years^29^ | Allowed | Heterosexual couples^1^ | Yes. ART with previously cryopreserved gametes is allowed.^1^ |
| 1. Turkey | By Law (Regulation) on Centres for Treatment (for medically assisted procreation) 31.03.2001 | Secondary^1,5,27^ | No data | No age limit for the use of stored oocytes and embryos^29^ | Oocytes, embryos, ovarian tissue for cancer patients and benign diseases.^29^ | Not allowed | The man can withdraw consent at any stage up to embryo’s implantation.^5^ | - Medication, doctor’s costs and tests up to a limit.^1^  - Not for couples or women with child(ren).  - Women till 39 years.  - Cryopreservation is not covered.^29^ | - 5 + 5 years for the oocytes with the possibility of extension.  - 10 years for the embryos and ovarian tissue^29^ | Allowed^27^ | Heterosexual couples^1^ | Previous gamete and gonadal tissue is not allowed for transgender^1^ |
| 1. UK | Human Fertilisation and Embryology Act 1990, amended 2008 | Primary^28^  Secondary^1,23,29^ | The term is not in use but there are references to storing gametes in case of treatment that can impair fertility. | - Women and men above 18 years of age.  - 50-55 years for the use of oocytes and embryos in practice ^29.^ | Oocytes, embryos, ovarian tissue for cancer patients and benign diseases.^29^ | - Allowed.  - The man needed to consent (and did not withdraw) in written form for the use of his sperm after his death. | - Consent is required.  - Gametes can be kept without consent when:  A) gametes are lawfully taken from a patient below 18 years,  B) medical practitioner certifies that the patient is about to undergo medical treatment that is likely to cause fertility impairment and the storage of gametes is in patient’s best interest.^28^ | - Public funds from the National Health Service (NHS), the Clinical Commissioning Groups (CCGs) and the Local Commissioning Groups (LCGs) and foundations.^23^  - Cryopreservation is covered.^29^  - Limit on female BMI.^1^ | - 10 years for embryos; can be renewed^28^  - For sperm longer than 10 years with consent.^28^  - Oocytes up to 10 years with the possibility of extension^29^ | - Allowed.  - Women can store eggs for non-medical reasons till 10 years, but as embryos - up to 55 years.^28^ | Heterosexual and lesbian couples and Single women^1^ | Yes. ART with previously cryopreserved gametes is allowed.^1^ |
| 1. Ukraine | 1) Decree of the Ministry of Health dtd 4 February 1997  2) Family Code of Ukraine dtd 1 January 2004  3) Decree N52/5 of the Ministry of Justice dtd 18 October 2000^9^ | Secondary^1,9,29^ | No data | - Women and men above 18 years of age.  - The age limit for use of stored material is defined by infertility specialist^29^ | Oocytes, embryos, ovarian tissue for cancer patients and benign diseases.^29^ | Allowed (March 2024) | No data | - ART is funded.  - Women till 40 years.  - Patient contributions for medication and tests.^1^  - Cryopreservation is not covered.^29^ | No limit both for oocytes and for embryos storage^29^ | Allowed^18^ | Heterosexual couples and Single women^1^ | Yes. Cryopreservation of oocytes, embryos, and ovarian tissue is allowed^29^. |
| EU-regulation | REGULATION (EU) 2024/1938 OF THE EUROPEAN PARLIAMENT AND OF THE COUNCIL of 13 June 2024  on standards of quality and safety for substances of human origin intended for human application and repealing Directives 2002/98/EC and 2004/23/EC | Primary^30^ | Definition of 'preservation of fertility' as the process of saving or protecting a person’s reproductive substance of human origin intended to be used later in that person’s life (Art.3 p.17). | Usage of preserved material should follow established standards (Art.60). | Sperm, oocytes, ovarian and testicular tissue | Considered for donated substances upon explicit authorization (consent in accordance with national legislation) to be used after death. | - The term is defined as permission given freely without coercion for an action affecting the person.  - Standards of information to be provided prior to consent (Art.55). | No texts. | Not specified. | No texts. | No texts. | No texts. |

^1^Calhaz-Jorge C, De Geyter CH, Kupka MS, Wyns C, Mocanu E, Motrenko T, Scaravelli G, Smeenk J, Vidakovic S, Goossens V. Survey on ART and IUI: legislation, regulation, funding and registries in European countries: The European IVF-monitoring Consortium (EIM) for the European Society of Human Reproduction and Embryology (ESHRE). Hum Reprod Open. 2020 Feb 6;2020(1):hoz044. doi: 10.1093/hropen/hoz044

^2^Gabriel Hofer-Ranz (2022) Reproduktion auf Eis gelegt? Ethische Aspekte von Social Egg Freezing · Nomos, Baden-Baden, 315 Seiten

^3^Balatinec I. Austrian Legislation and Jurisprudence on Medically Assisted Reproduction (2021). Available from: <https://medlawlab.web.auth.gr/wp-content/uploads/2021/12/Austrian-Legislation-and-Jurisprudence-on-Medically-Assisted-Reproduction.pdf>

^4^Pennings G. Decision-making authority of patients and fertility specialists in Belgian law. REPRODUCTIVE BIOMEDICINE ONLINE. 2007;15(1):19–23.

^5^Evans v. the United Kingdom, no. 6339/05, ECHR, 7 March 2006

^6^Bulgarian Parliament. Bulgarian Health Act. Announcement, SG No. 70 of 10.08.2004, in force since 1.01.2005. (2023) <https://lex.bg/laws/ldoc%20/2135489147> [Accessed February 15, 2025]

^7^Ministry of Health. Order No. H-2 of July 12, 2023 for assisted reproduction activities. (2023). <https://dv.parliament.bg/DVWeb/showMaterialDV.jsp;jsessionid=AA56A9BDF88EABB537C36EA841C41833?idMat=197485> [Accessed February 15, 2025]

^8^Busardò FP, Gulino M, Napoletano S, Zaami S, Frati P. The evolution of legislation in the field of Medically Assisted Reproduction and embryo stem cell research in European union members. Biomed Res Int. 2014;2014:307160. doi: 10.1155/2014/307160

^9^Council of Europe. Committee on Bioethics (DH-BIO). Replies by the member States to the questionnaire on access to medically assisted procreation (MAP), on the right to know about their origin for children born after MAP and on surrogacy. Strasbourg, 16 August 2023 Available from: <https://rm.coe.int/inf-2023-9-map-july-2023-replies-e/1680ad344f>

^10^Pejřilová v. the Czech Republic, no. 14889/19, ECHR, 8 March 2023

^11^ LOI n° 2021-1017 du 2 août 2021 relative à la bioéthique. Légifrance - Journal officiel électronique authentifié n° 0178 du 03/08/2021 Available from: <https://www.legifrance.gouv.fr/jorf/id/JORFTEXT000043884384>

^12^De Proost M, Johnston M. The revision of the French bioethics law and the questions it raises for the future of funding for egg freezing. Reprod Biomed Online. 2022 Apr;44(4):591-593. doi: 10.1016/j.rbmo.2021.12.002

^13^Labrosse J, Grynberg M. Fertility of tomorrow: Are there any restrictions left? Ann Endocrinol (Paris). 2022 Jun;83(3):207-209. doi: 10.1016/j.ando.2022.04.011

^14^Agopiantz M, Dap M, Martin E, Meyer L, Urwicz A, Mougel R, Malmanche H. Assisted reproductive technology in France: The reproductive rights of LGBT people. J Gynecol Obstet Hum Reprod. 2023 Dec;52(10):102690. doi: 10.1016/j.jogoh.2023.102690

^15^Pawłowski P, Ziętara KJ, Michalczyk J, Fryze M, Buchacz A, Zaucha-Prażmo A, Zawitkowska J, Torres A, Samardakiewicz M. Fertility Preservation in Children and Adolescents during Oncological Treatment-A Review of Healthcare System Factors and Attitudes of Patients and Their Caregivers. Cancers (Basel). 2023 Sep 2;15(17):4393. doi: 10.3390/cancers15174393

^16^Act on the Protection of Embryos. Federal Law Gazette, Part I, No. 69, issued in Bonn, 19th December 1990, page 2746 Available from: <https://www.bundesgesundheitsministerium.de/fileadmin/Dateien/3_Downloads/Gesetze_und_Verordnungen/GuV/E/ESchG_EN_Fassung_Stand_10Dez2014_01.pdf>

^17^ESHRE. Comparative Analysis of Medically Assisted Reproduction in the EU: Regulation and Technologies (SANCO/2008/C6/051). Final Report. 2008. Grimbergen: ESHRE Central Office. p.166. Available from: <https://health.ec.europa.eu/document/download/3abc7b2a-cc86-4eac-8036-db18a34cb586_en>

^18^Kynigopoulou S, Matsas A, Tsarna E, Christopoulou S, Panagopoulos P, Bakas P, Christopoulos P. Egg Cryopreservation for Social Reasons—A Literature Review. *Healthcare*. 2024; 12(23):2421. <https://doi.org/10.3390/healthcare12232421>

^19^Health (Assisted Human Reproduction) Act No.18/2024. Available from: <https://www.irishstatutebook.ie/eli/2024/act/18/enacted/en/pdf>

^20^Code of the Republic of Kazakhstan “On People’s Health and Healthcare System” No. 360-VI/July 7, 2020 Available from Adilet: Legal information system of Regulatory Legal Acts of Republic of Kazakhstan: <https://adilet.zan.kz/eng/docs/K2000000360>

^21^Republic of Moldova Parliament. Law No. 138 of 15.06.2012 regarding reproductive health. Available from: <https://natlex.ilo.org/dyn/natlex2/natlex2/files/download/111901/MDA-111901%20(EN).pdf>

^22^Micković D, Ristov A. Biomedical assisted fertilization in Macedonia, Serbia and Croatia ethical and legal aspects. SEE Law Journal, 2014, 1(1):21-34. Available from: <https://www.seelawschool.org/pdf/1_Biomedical_Assisted_Fertilization_in_Macedonia_Serbia_and_Croatia_Ethical_and_Legal_Aspects.pdf>

^23^Pawłowski P, Ziętara KJ, Michalczyk J, Fryze M, Buchacz A, Zaucha-Prażmo A, Zawitkowska J, Torres A, Samardakiewicz M. Fertility Preservation in Children and Adolescents during Oncological Treatment-A Review of Healthcare System Factors and Attitudes of Patients and Their Caregivers. Cancers (Basel). 2023 Sep 2;15(17):4393. doi: 10.3390/cancers15174393

^24^Wennberg AL. Social freezing of oocytes: a means to take control of your fertility. Ups J Med Sci. 2020 May;125(2):95-98. doi: 10.1080/03009734.2019.1707332.

^25^Federal Act on Medically Assisted Reproduction (Reproductive Medicine Act, RMA) of 18 December 1998 (Status as of 1 July 2023). Available from: <https://www.fedlex.admin.ch/eli/cc/2000/554/en>

^26^Rimon-Zarfaty N, Kostenzer J, Sismuth LK, de Bont A. Between "Medical" and "Social" Egg Freezing : A Comparative Analysis of Regulatory Frameworks in Austria, Germany, Israel, and the Netherlands. J Bioeth Inq. 2021 Dec;18(4):683-699. doi: 10.1007/s11673-021-10133-z.

^27^Kılıç A, Göçmen İ. Fate, morals and rational calculations: Freezing eggs for non-medical reasons in Turkey. Soc Sci Med. 2018 Apr;203:19-27. doi: 10.1016/j.socscimed.2018.03.014.

^28^Human Fertilisation and Embryology Act 1990. Available from: <https://www.legislation.gov.uk/ukpga/1990/37#commentary-key-3b876889c220097e5c8299fd22ab2954>

^29^ ESHRE Female Fertility Preservation Guideline Development Group. Female Fertility Preservation. Guideline of the European Society of Human Reproduction and Embryology, 2020. European Society of Human Reproduction and Embryology, Strombeek-Bever, Belgium, p.185. Accessible in Internet at: <https://www.eshre.eu/Guidelines-and-Legal/Guidelines/Female-fertility-preservation>

^30^European Parliament, Council of the European Union. REGULATION (EU) 2024/1938 OF THE EUROPEAN PARLIAMENT AND OF THE COUNCIL of 13 June 2024 on standards of quality and safety for substances of human origin intended for human application and repealing Directives 2002/98/EC and 2004/23/EC. Official Journal of the European Union L series 17.7.2024. Available from: <http://data.europa.eu/eli/reg/2024/1938/oj>
