# Supplementary material for: Ethical analysis of the European normative framework on fertility preservation
Source: BMC Med Ethics. 2026 Feb 2;27:38. doi: 10.1186/s12910-026-01393-8 (PMC12930747; doi:10.1186/s12910-026-01393-8)
Supplement: Supplementary file 3 — Supplementary Material 3. [file 12910_2026_1393_MOESM3_ESM.docx]

Additional file 3. Overview of results of thematic analysis of guidelines regarding fertility preservation

| Guideline | Organization Country | Source | Topic I –  Definitions of fertility preservation | Topic II -  Age limits for fertility preservation/ ART access | Topic III –  Type of preserved reproductive biomaterial | Topic IV –  Posthumous usage | Topic V -  Informed consent | Topic VI –  State/Health insurance funding | Topic VII -  Storage period | Topic VIII -  Social egg freezing | Topic IX –  Requirements for partnership status | Topic X -  Special provisions for transgender individuals |
| --- | --- | --- | --- | --- | --- | --- | --- | --- | --- | --- | --- | --- |
| 1. NICE “Fertility problems assessment and treatment”, 2017 | National Institute for Health and Care Excellence, UK | NICE. Fertility problems:assessment and treatment. Clinical guideline [CG156], Published:20 February 2013, Last updated: 06 September 2017. Available from: <https://www.nice.org.uk/guidance/cg156> | Term in use but no definition | No full ART cycles after 40 years.  With limits between 40 and 42 years. | Oocyte and embryo cryopreservation for women of reproductive age. | No texts | - Focus on information provision.  - Independent counselling.  - No instruction on the process. | No texts | Extension of cryopreserved sperm beyond 10 years to men who remain at high risk of infertility | No texts | No texts | No texts |
| 1. Standards of Care for the Health of Transgender and Gender Diverse People, Version 8, 2022 | World Professional  Association for Transgender Health’s (WPATH) | E. Coleman et al. (2022) Standards of Care for the Health of Transgender and Gender Diverse People, Version 8, International Journal of Transgender Health, 23:sup1, S1-S259, DOI:  ^10.1080/26895269.2022.2100644^ | Term in use but no definition | Barriers mentioned but no age limit. | Cryopreservation of sperm, oocytes, ovarian tissue before gender assignment treatment. | No texts | Fertility counselling. | Discussed in general for gender-affirming treatment, not specifically for fertility preservation. | No texts | No texts | No texts | Yes since it is a specifically-targeted document. |
| 1. EUropean REcommendations for female FERtility preservation (EU-REFER): A joint collaboration between oncologists and fertility specialists, 2019 | European oncofertility experts | Dolmans M et al. A. EUropean REcommendations for female FERtility preservation (EU-REFER): A joint collaboration between oncologists and fertility specialists. Crit Rev Oncol Hematol. 2019 Jun;138:233-240. doi: 10.1016/j.critrevonc.2019.03.010 | Term in use, methods for fertility preservation underlined, no separate definition | 43 years for oocyte freezing and 36 years for ovarian tissue freezing | Embryo, oocyte and ovarian tissue cryopreservation. | No texts | Yes.  - Oncofertility consultation checklist provided.  - Multidisciplinary team  - Several discussions  - In person or videoconference  - Also, post-treatment | Discussed and comparative data for European countries are provided. | No texts | No texts | No texts | No texts |
| 1. The Oncology Association of Bosnia and Herzegovina’s recommendations for fertility preservation in oncologic patients, 2022 | Oncology Association of Bosnia and Herzegovina | Cerić T et al. , The Oncology Association of Bosnia and Herzegovina's recommendations for fertility preservation in oncologic patients. Bosn J Basic Med Sci. 2022 Sep 16;22(5):646-650. doi: 10.17305/bjbms.2021.6977. | Term in use, methods for fertility preservation underlined, no separate definition | No texts | Embryo, oocyte, sperm, testicular and ovarian tissue cryopreservation. | No texts | Term not in use.  - Timely discussion before treatment  - Multidisciplinary counselling | Only in private centers. Lack of health insurance coverage. | No texts | No texts | No texts | No texts |
| 1. Fertility Preservation for Patients with Malignant Disease. Guideline of the DGGG, DGU and DGRM (S2k-Level, AWMF Registry No. 015/082, November 2017) – Recommendations and Statements for Girls and Women, 2017 | Published by the German  Society of Gynecology and Obstetrics (DGGG) and coordinated  with the German Society of Urology (DGU) and the  German Society of Reproductive Medicine (DGRM) | Dittrich R et al. Fertility Preservation for Patients with Malignant Disease. Guideline of the DGGG, DGU and DGRM (S2k-Level, AWMF Registry No. 015/082, November 2017) - Recommendations and Statements for Girls and Women. Geburtshilfe Frauenheilkd. 2018 Jun;78(6):567-584. doi: 10.1055/a-0611-5549. | Term in use, methods for fertility preservation underlined, no separate definition | Reproductive age | Cryopreservation of fertilized and unfertilized oocytes, sperm, ovarian and testicular tissue. | No texts | Yes.  - Timely discussion before treatment | No texts | No texts | No texts | No texts | No texts |
| 1. ESGO/ESTRO/ESP Guidelines for the management of patients with endometrial carcinoma, 2021 | European Society of Gynaecological Oncology (ESGO), the European SocieTy for Radiotherapy & Oncology (ESTRO) and the  European Society of Pathology (ESP) | Concin N et al. ESGO/ESTRO/ESP guidelines for the management of patients with endometrial carcinoma. Int J Gynecol Cancer. 2021 Jan;31(1):12-39. doi: 10.1136/ijgc-2020-002230 | Term in use, no separate definition | No texts | Oocyte and ovarian cryopreservation | No texts | Term not in use.  - Multidisciplinary discussions with clinical focus; patient involvement is not specified | No texts | No texts | No texts | No texts | No texts |
| 1. ESGO/ESHRE/ESGE Guidelines for the fertility-sparing treatment of patients with endometrial carcinoma, 2023 | European Society of Gynaecological Oncology (ESGO), the European  Society of Human Reproduction and Embryology (ESHRE) and the  European Society for Gynecological Endoscopy (ESGE) | Rodolakis A et al. ESGO/ESHRE/ESGE Guidelines for the fertility-sparing treatment of patients with endometrial carcinoma. Hum Reprod Open. 2023 Feb 6;2023(1):hoac057. doi: 10.1093/hropen/hoac057 | Term in use, no separate definition | No texts | No texts | No texts | Term not in use.  - Consultation with fertility specialists before fertility-sparing treatment  - Multidisciplinary counselling for all patients with a pregnancy wish  - Advice on conception as soon as complete treatment response is achieved. | No texts | No texts | No texts | No texts | No texts |
| 1. Female Fertility Preservation Guideline of the European Society of Human Reproduction and Embryology, 2020 | European Society of Human Reproduction in Embryology (ESHRE) | ESHRE Female Fertility Preservation Guideline Development Group. Female Fertility Preservation. Guideline of the European Society of Human Reproduction and Embryology, 2020. European Society of Human Reproduction and Embryology, Strombeek-Bever, Belgium, p.185. Accessible in Internet at: <https://www.eshre.eu/Guidelines-and-Legal/Guidelines/Female-fertility-preservation> | Yes | Age limits for the usage of the stored reproductive material. | Oocyte, embryo, and ovarian tissue cryopreservation | No texts | Yes.  - Information needs and provision.  - Decision aids to support patients and tools to support clinicians.  - Also, post-treatment counselling. | Considered as extrinsic factor influencing the patients’ assessment for fertility preservation and as a issue with oocyte cryopreservation for age-related fertility loss. | Yes.  Depends on country's regulation. | Yes.  Oocyte cryopreservation for age-related fertility loss. | Available for some countries in regard to access and reimbursement of fertility preservation, e.g. in Turkey only married couples can preserve embryos. | Yes.  Specific care for transgender men and availability of cryopreservation options. |
| 1. Fertility preservation and post-treatment pregnancies in post-pubertal cancer patients: ESMO Clinical Practice Guidelines, 2020 | European Society for Medical Oncology (ESMO) | Lambertini M et al. ESMO Guidelines Committee. Fertility preservation and post-treatment pregnancies in post-pubertal cancer patients: ESMO Clinical Practice Guidelines†. Ann Oncol. 2020 Dec;31(12):1664-1678. doi: 10.1016/j.annonc.2020.09.006 | Term in use, methods for fertility preservation underlined, no separate definition | - Oocyte or embryo cryopreservation for women preferably up to 40 years of age;  -Ovarian tissue cryopreservation for women up to 36 years of age. | Oocyte, embryo and ovarian tissue cryopreservation. Sperm cryopreservation. | No texts | Term not in use.  - All cancer patients of reproductive age should receive complete oncofertility counselling.  - Written information and online resources additionally.  - Timely referral to fertility specialist | To guarantee access to fertility preservation for every cancer patient, universal insurance coverage should be implemented. | No specific period is mentioned but long-term sperm storage is discussed as not worsening sperm quality. | No texts | Women choosing to store embryos created with  their partner’s sperm should be advised on the joint property of embryos and issues with their usage in the event of broken the relationship . | No texts |
| 1. ESO-ESMO fifth international consensus guidelines for breast cancer in young women (BCY5), 2022 | European School  of Oncology (ESO) and European Society of Medical  Oncology (ESMO) | Paluch-Shimon S et al. ESO-ESMO fifth international consensus guidelines for breast cancer in young women (BCY5). Ann Oncol. 2022 Nov;33(11):1097-1118. doi: 10.1016/j.annonc.2022.07.007 | Term in use, no separate definition | For ovarian suppression recommended age limit of 35 years. | Oocyte cryopreservation | No texts | - Informed decision about the main disease.  - All young women should be informed about approved fertility preservation options and if interested in, referred for specialist counseling before the commencement of any  therapy. | Young breast cancer survivors experience potential financial toxicity following  diagnosis. | No texts | No texts | No texts | Yes. Cancer registries should include TG/NB  identities, and further education and research is needed to fill the gap in information and counseling in this population. |
| 1. ULAR recommendations for women's health and the management of family planning, assisted reproduction, pregnancy and menopause in patients with systemic lupus erythematosus and/or antiphospholipid syndrome, 2017 | European League Against  Rheumatism (EULAR) | Andreoli L et al. EULAR recommendations for women's health and the management of family planning, assisted reproduction, pregnancy and menopause in patients with systemic lupus erythematosus and/or antiphospholipid syndrome. Ann Rheum Dis. 2017 Mar;76(3):476-485. doi: 10.1136/annrheumdis-2016-209770 | Term in use, methods for fertility preservation underlined, no separate definition | No limits to access. | Cryopreservation of ovarian tissue, oocytes, embryos are mentioned as poorly investigated for these patients. | No texts | Term not in use.  - The preservation of fertility should be mentioned while counselling about lifestyles and considered in the treatment choice. | No texts | No texts | No texts | No texts | No texts |
| 1. Practical recommendations for fertility preservation in women by the FertiPROTEKT network. Part I: Indications for fertility preservation, 2018 | Ferti-  PROTEKT network: a network and society of physicians  and biologists specializing in fertility preservation in Germany,  Austria and parts of Switzerland | Schüring AN et al. Practical recommendations for fertility preservation in women by the FertiPROTEKT network. Part I: Indications for fertility preservation. Arch Gynecol Obstet. 2018 Jan;297(1):241-255. doi: 10.1007/s00404-017-4594-3. | Term in use, fertility preservation measures for different diagnoses are presented, no separate definition | Fertility preservation is recommended in women with breast cancer with a good prognosis, with a moderate to high Premature Ovarian Insufficiency risk and/or age up to 35 years. | Cryopreservation of oocytes and ovarian tissue. | No texts | Term used in relation to treatment decisions but no details about the content of information or the process itself. | No texts | No texts | No texts | No texts | No texts |
| 1. Practical recommendations for fertility preservation in women by the FertiPROTEKT network. Part II: fertility preservation techniques, 2018 | Ferti-  PROTEKT network | von Wolff M et al. Practical recommendations for fertility preservation in women by the FertiPROTEKT network. Part II: fertility preservation techniques. Arch Gynecol Obstet. 2018 Jan;297(1):257-267. doi: 10.1007/s00404-017-4595-2 | Term in use, methods for fertility preservation underlined, no separate definition | The upper age limit for cryopreservation of ovarian tissue is 35 years or 38 years for women with a high ovarian reserve. | Cryopreservation of oocytes and ovarian tissue. | No texts | Term used but no details on the content of information or the process itself. | No texts | In the explanation of ovarian tissue freezing, the possibility of indefinite storage at -196°C is mentioned. | No texts | It should be noted that fertilized oocytes can only be transferred to the woman after the consent  of both partners. | No texts |
| 1. Borderline ovarian tumors: French guidelines from the CNGOF. Part 2. Surgical management, follow-up, hormone replacement therapy, fertility management and preservation, 2021 | The “Collège National des Gynécologues et Obstétriciens Français” (CNGOF) | Bourdel N et al. Borderline ovarian tumors: French guidelines from the CNGOF. Part 2. Surgical management, follow-up, hormone replacement therapy, fertility management and preservation. J Gynecol Obstet Hum Reprod. 2021 Jan;50(1):101966. doi: 10.1016/j.jogoh.2020.101966 | Term in use, techniques are mentioned, no separate definition | Only medical criteria related to risk of ovarian stimulation | Oocyte cryopreservation after conservative surgical treatment | No texts | Term not in use.  - It is recommended that patients are provided with full information on the risk of a decrease in ovarian reserve associated with the surgical treatment of the main disease. | No texts | No texts | No texts | No texts | No texts |
| 1. Management of epithelial cancer of the ovary, fallopian tube, primary peritoneum. Long text of the joint French clinical practice guidelines issued by FRANCOGYN, CNGOF, SFOG, GINECO-ARCAGY, endorsed by INCa. (Part 2: systemic, intraperitoneal treatment, elderly patients, fertility preservation, follow-up), 2019 | The French research group for oncologic gynecologic surgery (FRANCOGYN), the French national college of gynecologists and obstetricians (CNGOF), the French society of gynecologic oncology (SFOG), and the national investigators' group for studies in ovarian and breast cancer (GINECO-ARCAGY) | Lavoue V et al. Management of epithelial cancer of the ovary, fallopian tube, primary peritoneum. Long text of the joint French clinical practice guidelines issued by FRANCOGYN, CNGOF, SFOG, GINECO-ARCAGY, endorsed by INCa. (Part 2: systemic, intraperitoneal treatment, elderly patients, fertility preservation, follow-up). J Gynecol Obstet Hum Reprod. 2019 Jun;48(6):379-386. doi: 10.1016/j.jogoh.2019.03.018 | Term in use, no separate definition | No | Ovarian tissue freezing is mentioned in the context of lack of evidence to justify it for women with epithelial ovarian cancers. | No texts | Term not in use.  - Women who wish fertility preservation must be informed that there is a risk of recurrence. | No texts | No texts | No texts | No texts | No texts |
| 1. EMAS position statement: Fertility preservation, 2013 | European Menopause and Andropause Society (EMAS) | Mintziori G et al. EMAS position statement: Fertility preservation. Maturitas. 2014 Jan;77(1):85-9. doi: 10.1016/j.maturitas.2013.10.010 | Term in use, no separate definition | Women of reproductive age, pre-pubertal boys and girls | Testicular and ovarian tissue cryopreservation. Cryopreservation of oocytes and embryos. | No texts | Term not in use.  - All women of reproductive age or younger should be thoroughly informed before the initiation of any gonadotoxic treatment about all available options for fertility preservation.  - In the case of pre-pubertal boys and girls the discussion should be done together with the patients. | No texts | No texts | No texts | No texts | No texts |
| 1. Fertility preservation for female patients with childhood, adolescent, and young adult cancer: recommendations from the PanCareLIFE Consortium and the International Late Effects of Childhood Cancer Guideline Harmonization Group, 2021 | EU-funded project, PanCareLIFE, in collaboration with the International Late Effects of Childhood Cancer Guideline Harmonization Group (IGHG) | Mulder RL et al. Fertility preservation for female patients with childhood, adolescent, and young adult cancer: recommendations from the PanCareLIFE Consortium and the International Late Effects of Childhood Cancer Guideline Harmonization Group. Lancet Oncol. 2021 Feb;22(2):e45-e56. doi: 10.1016/S1470-2045(20)30594-5. | Term in use, techniques are mentioned, no separate definition | No | Oocytes, embryo and ovarian tissue cryopreservation | No texts | Yes.  - It is important to inform patients and their families about the potential benefits, harms, costs, and logistics associated with fertility preservation for decisions to be well informed.  - Counselling about options for fertility preservation and alternative family planning. | No texts | No texts | No texts | No texts | No texts |
| 1. Fertility preservation for male patients with childhood, adolescent, and young adult cancer: recommendations from the PanCareLIFE Consortium and the International Late Effects of Childhood Cancer Guideline Harmonization Group, 2021 | EU-funded project, PanCareLIFE, in collaboration with the International Late Effects of Childhood Cancer Guideline Harmonization Group (IGHG) | Mulder RL et al. Fertility preservation for male patients with childhood, adolescent, and young adult cancer: recommendations from the PanCareLIFE Consortium and the International Late Effects of Childhood Cancer Guideline Harmonization Group. Lancet Oncol. 2021 Feb;22(2):e57-e67. doi: 10.1016/S1470-2045(20)30582-9 | Term in use, techniques are mentioned, no separate definition | No | Sperm and testicular tissue cryopreservation | No texts | - Shared decision-making between health-care providers and patients and their families is essential when decisions are made about fertility preservation.  - It is important to inform patients and their families about the potential benefits, harms, costs, and logistics that are associated with fertility preservation. | No texts | Length of storage is mentioned as a factor affecting the quality of sperm but not specified in the number of years. | No texts | No texts | No texts |
| 1. Communication and ethical considerations for fertility preservation for patients with childhood, adolescent, and young adult cancer: recommendations from the PanCareLIFE Consortium and the International Late Effects of Childhood Cancer Guideline Harmonization Group, 2021 | EU-funded project, PanCareLIFE, in collaboration with the International Late Effects of Childhood Cancer Guideline Harmonization Group (IGHG) | Mulder RL et al. Communication and ethical considerations for fertility preservation for patients with childhood, adolescent, and young adult cancer: recommendations from the PanCareLIFE Consortium and the International Late Effects of Childhood Cancer Guideline Harmonization Group. Lancet Oncol. 2021 Feb;22(2):e68-e80. doi: 10.1016/S1470-2045(20)30595-7 | Term in use. Short definition: Fertility preservation aims to give patients future reproductive autonomy. | Age as a factor for participation in discussion-making: Most parents considered their 12 to 18 years old child as capable to participate in decision-making, some even for 7 to 12 years old. | Preservation of gametes and tissues | Posthumous usage is mentioned and the potential disagreement among family members about gamete disposal in the event of a patient’s death is underlined. | Yes.  - Two-stage consent process.  - Specific attention to accompanying ethical issues.  - Age-appropriate information and online educational resources.  - Private conversation with the patient and a separate conversation with the family  - Timely counselling | - Cost of procedures as a barrier related to the healthcare provider and institution.  - Referrals for charity or other programmes that might help to offset costs are recommended. | No texts | No texts | No texts | No texts |
| 1. Multidisciplinary consensus on the criteria for fertility preservation in cancer patients, 2021 | Spanish Society of Medical Oncology (SEOM), the Spanish Fertility  Society (SEF), the Spanish Society of Haematology and  Haemotherapy (SEHH), the Spanish Society of Paediatric  Haematology and Oncology (SEHOP) and the Spanish  Society of Radiation Oncology (SEOR) | Santaballa A et al. Multidisciplinary consensus on the criteria for fertility preservation in cancer patients. Clin Transl Oncol. 2022 Feb;24(2):227-243. doi: 10.1007/s12094-021-02699-2. | Yes. Fertility preservation  techniques will be performed in patients with a possible  risk of loss of their reproductive capacity associated with exposure to gametotoxic treatments or pathological  processes with a proven risk of premature ovarian failure or primary testicular failure. | Females above 2 years and up to 35-40 years with a preserved ovarian reserve. | Oocyte, semen and ovarian cryopreservation. | Yes.  - It is necessary to inform patients of the legal conditions regulating the use of their gametes in the event of death. In such cases, for semen to be used to fertilise the patient’s wife or partner, there must be prior consent in a public deed or  will, and the semen must be used within twelve months of the patient’s death.  - Consent  is presumed to have been given when an assisted reproduction process is initiated prior to the death of a male partner. | Yes.  - Specific consent must be obtained from patients with medical indications who express interest in fertility preservation.  - In minors, consent of the child and his or her parents or legal guardians is required. | No texts | No specification but a signed consent is required to remove cryopreserved samples and to destroy them. | No texts | In the case of a married man, the birth  of a child conceived after his death shall produce  the legal effects derived from the marital affiliation. | No texts |
| 1. SEOM Clinical Guideline of fertility preservation and reproduction in cancer patients, 2016 | Spanish Society of Medical Oncology (SEOM) | Muñoz M et al. SEOM Clinical Guideline of fertility preservation and reproduction in cancer patients (2016). Clin Transl Oncol. 2016 Dec;18(12):1229-1236. doi: 10.1007/s12094-016-1587-9. | Term in use, techniques are mentioned, no separate definition | Reproductive age | Oocytes, sperm, embryo, ovarian tissue, and testicular tissue cryopreservation | No texts | Yes.  - For prepubertal patients also assent.  - No details about the content of the information or the process itself. | No texts | No texts | No texts | No texts | No texts |
| 1. Fertility Preservation in Oncological and Non-Oncological Diseases. A Practical Guide. 2020 | The book is intended to be a practical guide for the indication and implementation  of fertility preservation measures for all those who belonged to the German Austrian  Swiss network FertiPROTEKT | von Wolff M., Nawroth F. Fertility preservation in Oncological and Non-Oncological DIseases. A Practical Guide. 2020. Sham, Switzerland: Springer | Term in use, techniques are mentioned, no separate definition | - Up to 35 years for cryopreservation of ovarian tissue;  - Up to 40 years for fertility preservation generally for women  - No age limit for men | Oocytes, sperm, ovarian and testicular tissue | In countries where the use of cryopreserved semen samples is permitted posthumously, cryopreservation is also recommended for men within the scope of a hazardous activity, e.g. military service. In principle, it is possible for every man to create a fertility reserve (“social freezing”). | Yes.  - For women, before the cryopreservation of oocytes or ovarian tissue written consent should be obtained from the patient or legal guardian concerning how long the material will be kept frozen, at what intervals the consent will be renewed and the destroying of material in the event of patient’s death.  - Fertilised eggs can only be transferred to the woman with the consent of both partners. | The need of justification of costs for fertility preservation treatment to the health insurance company addressed. | Several years but not specified. Studies over deterioration of semen quality depending on storage length are cited. | No texts | A separation in the couple during the time of fertilized eggs storage means that the woman would no longer have the option of retransferring them. To avoid this risk, it is recommended to store unfertilized oocytes 50% or 100% to guarantee woman’s independence. | Yes. Separate chapter on counselling of transgender on fertility preservation. |
| 1. Management of endometriosis: CNGOF/HAS clinical practice guidelines – Short version, 2018 | French National Authority for Health (HAS) and the French College of Gynaecologists and Obstetricians (CNGOF) | Collinet P et al. Management of endometriosis: CNGOF/HAS clinical practice guidelines - Short version. J Gynecol Obstet Hum Reprod. 2018 Sep;47(7):265-274. doi: 10.1016/j.jogoh.2018.06.003 | Term in use, methods not presented, no separate definition | No | Oocyte retrieval for IVF but no freezing | No texts | - Informed decision for therapy of main disease but not specific for fertility preservation.  - Printed information is encouraged  - Physicians are invited to fully inform women with endometriosis about fertility (Expert Consensus). | No texts | No texts | No texts | Partners of women are referred in relation to receiving information. | No texts |
| 1. FIGO statement: Fertility preservation, 2023 | International Federation of Gynecology and Obstetrics (FIGO) | Henry L et al. FIGO statement: Fertility preservation. Int J Gynaecol Obstet. 2023 Dec;163(3):790-794. doi: 10.1002/ijgo.15187. | Yes. Short definition: Fertility preservation may allow patients with cancer and other patients to build families  with their own genetic makeup. | Reproductive age | Oocytes, sperm, testicular and ovarian tissue, embryo cryopreservation | No texts | The patient, ideally, should be referred to a multidisciplinary team experienced in fertility preservation procedures. | No texts | No texts | No texts | As a requirement to offer embryo cryopreservation, the woman should be in a stable relationship and informed about the legal joint ownership with the male over the embryos. | Yes. It is important that transgender patients are managed in a multidisciplinary manner by a specialized team so that they can make an informed decision in relation to fertility preservation. |
| 1. Management of Germ Cell Tumours of the Testis in Adult Patients. German Clinical Practice Guideline. Part I: Epidemiology, Classification, Diagnosis, Prognosis, Fertility Preservation, and Treatment Recommendations for Localized Stages, 2021 | The German Society of Urology (DGU) together  with interdisciplinary German  Testicular Cancer Study Group (GTCSG) of the German  Cancer Society | Kliesch S et al. Management of Germ Cell Tumours of the Testis in Adult Patients. German Clinical Practice Guideline Part I: Epidemiology, Classification, Diagnosis, Prognosis, Fertility Preservation, and Treatment Recommendations for Localized Stages. Urol Int. 2021;105(3-4):169-180. doi: 10.1159/000510407. | Term in use, no separate definition | No texts | Sperm cryopreservation | No texts | Term not in use.  - Cryopreservation to be offered before any treatment. | No texts | No texts | Not applicable | No texts | No texts |
| 1. Fertility preservation for medical reasons in girls and women: British fertility society policy and practice guideline, 2018 | British Fertility Society | Yasmin E et al. British Fertility Society. Fertility preservation for medical reasons in girls and women: British fertility society policy and practice guideline. Hum Fertil (Camb). 2018 Apr;21(1):3-26. doi: 10.1080/14647273.2017.1422297 | Term in use, methods represented, no separate definition | Reproductive age.  Up to 35 years for ovarian tissue cryopreservation. | Oocytes, embryo and ovarian tissue cryopreservation | No texts | Yes.  - Relevant informed consent form should be completed at each extension of the storage period.  - Consent of both partners for embryo cryopreservation; counseling should address the requirement for both partners to consent to the use of embryos in any future fertility treatment.  - Counselling as early as possible in cancer treatment pathway.  -Refer women to a trained counsellor both prior to fertility preservation and prior to use of stored material.  -Decision-making aids should be provided to support women’s fertility preservation decision-making. | Yes.  - Patients need to be made aware that subsequent NHS-funding for use of oocytes/embryos may depend on meeting criteria for fertility treatment at the time. | Yes.  - Initial storage maximum 10 years extendable by another 10 years up to 55 years. | No texts. Explicit statement that elective (‘social’) fertility preservation is outside the remit of the guideline, although  the technical considerations for oocyte freezing are the same. | As a requirement to offer embryo cryopreservation the woman should be in a stable relationship. | Yes.  - Discuss fertility preservation with people transitioning from female to male Gender.  - If appropriate, fertility preservation should be performed as early as possible  in the treatment pathway.  - Offer counselling, both prior to fertility preservation and prior to use of stored material. |
